# Association of *SLC6A4* methylation with long-term outcomes after stroke: focus on the interaction with suicidal ideation

**DOI:** 10.1038/s41598-021-81854-9

**Published:** 2021-02-01

**Authors:** Hee-Ju Kang, Eun-Hye Lee, Ju-Wan Kim, Sung-Wan Kim, Il-Seon Shin, Joon-Tae Kim, Man-Seok Park, Ki-Hyun Cho, Jung-Soo Han, In Kyoon Lyoo, Jae-Min Kim

**Affiliations:** 1 Department of Psychiatry, Chonnam National University Medical School, 160 Baekseoro, Dong-gu, Gwangju, 61669 Republic of Korea; 2 Department of Neurology, Chonnam National University Medical School, Gwangju, Republic of Korea; 3 Department of Biological Science, Konkuk University, 120 Neungdong-ro, Gwangjin-gu, Seoul, 05029 Republic of Korea; 4 Ewha Brain Institute, Graduate School of Pharmaceutical Sciences, and Department of Brain and Cognitive Sciences, Ewha W. University, Seoul, Republic of Korea

**Keywords:** Prognostic markers, Stroke

## Abstract

Serotonin (5-HT) plays an important role in cerebrovascular homeostasis and psychiatric disorders, including suicidality. Methylation of the serotonin transporter gene (*SLC6A4*) is associated with 5-HT expression. However, the prognostic roles of *SLC6A4* methylation and suicidal ideation (SI) in long-term outcomes of stroke have not been evaluated. We investigated the independent and interactive effects of *SLC6A4* methylation and SI immediately after stroke on long-term outcomes. Blood *SLC6A4* methylation status and SI based on the suicide item of the Montgomery–Åsberg Depression Rating Scale were assessed in 278 patients at 2 weeks after stroke. After the index stroke, cerebro-cardiovascular events by *SLC6A4* methylation status and SI were investigated over an 8–14-year follow-up period and using Cox regression models adjusted for a range of covariates. *SLC6A4* hypermethylation and SI within 2 weeks of stroke both predicted worse long-term outcomes, independent of covariates. A significant interaction effect of SI and the methylation status of CpG 4 on long-term stroke outcomes was also identified. The association between *SLC6A4* methylation and long-term adverse outcomes may be strengthened in the presence of SI within 2 weeks after stroke. Evaluation of methylation and SI status during the acute phase can be helpful when assessing stroke patients.

## Introduction

Stroke is a leading cause of disability and death. The main treatment goal of stroke management is to improve long-term outcomes by reducing the likelihood of stroke recurrence, other cardiovascular events, and mortality^1^. Considerable effort has been made to predict the long-term risk of poor vascular outcomes, including recurrent stroke, myocardial infarction, and vascular death, in stroke patients^2–4^.

Based on findings from animal and human studies, serotonin (5-HT) is a potential candidate for predicting stroke outcomes. Modulation of 5-HT has been reported to alter motor cortex excitability and promote motor recovery^5^. Also, 5-HT was shown to be associated with aspects of cardiovascular homeostasis, including platelet aggregation and monitoring of vascular tone, cerebrovascular function and cardiac function^6^. Against this background, selective serotonin reuptake inhibitors (SSRIs), which increase 5-HT levels in the synaptic cleft by blocking reuptake of 5-HT selectively through serotonin transporter, can improve neurogenesis and infarct volume^7^, thus functional recovery^8,9^, although some recent findings have been controversial^10,11^.

Serotonin transporter (5-HTT), the regulators of serotonin function in both the brain and the periphery, is encoded by the *solute carrier family 6 member 4* gene (*SLC6A4*) located on chromosome 17q11.1–17q12. The expression and function of 5-HTT are regulated by several functional polymorphisms including the 5-HTT-linked promoter region (5-HTTLPR) and a variable number of tandem repeats in the 5-HTT intron 2 (STin2 VNTR)^12^ and by epigenetic mechanisms such as DNA methylation^13^. In terms of *SLC6A4* polymorphisms, those related to lower expression of 5-HTT were associated with reduced 5-HT uptake in lymphoblast cells^14^ and blood platelets^15,16^, and a lower level of 5-HIAA (the main metabolite of 5-HT)^17^, although some studies have found no differences^16,18^. Considering the role of *SLC6A4* polymorphisms, two previous studies investigated the association between *SLC6A4* polymorphisms and short-term (within 6 months) stroke outcomes. They reported that stroke patients with the STin 12/10 polymorphism (lower 5-HTT expression) tended to attain better functional recovery in response to an SSRI than did those with STin 12/12 polymorphism (higher 5-HTT expression)^19^ and stroke patients with the low 5-HTT expression genotype showed poorer cognitive recovery in response to the placebo compared to the SSRI^20^. The effects of methylation of the *SLC6A4* gene on stroke outcomes have not been investigated, although hypermethylation of the *SLC6A4* gene was related to lower 5-HTT mRNA levels and brain serotonin synthesis^21^.

Furthermore, 5-HT is a crucial neurotransmitter related to suicide^22^ that has been investigated outside the stroke population^23^, to date, no studies have examined the serotonin level in stroke patients. Based on these findings, the function of serotonin can affect long-term outcomes in stroke patients directly by modulating cerebro-cardiovascular homeostasis and neurological recovery^19,24^ and perhaps indirectly through psychiatric symptoms such as suicidality. In this study, we investigated independent and interactive effects of the methylation status of the *SLC6A4* gene and suicidal ideation (SI) immediately after stroke (within 2 weeks) on the long-term outcomes (8–14 years) of patients who experienced cerebro-cardiovascular events (CCVEs), including recurrent stroke, myocardial infarction, and vascular death, using data from a longitudinal stroke cohort.

## Methods

### Study overview and participants

**Figure 1:**
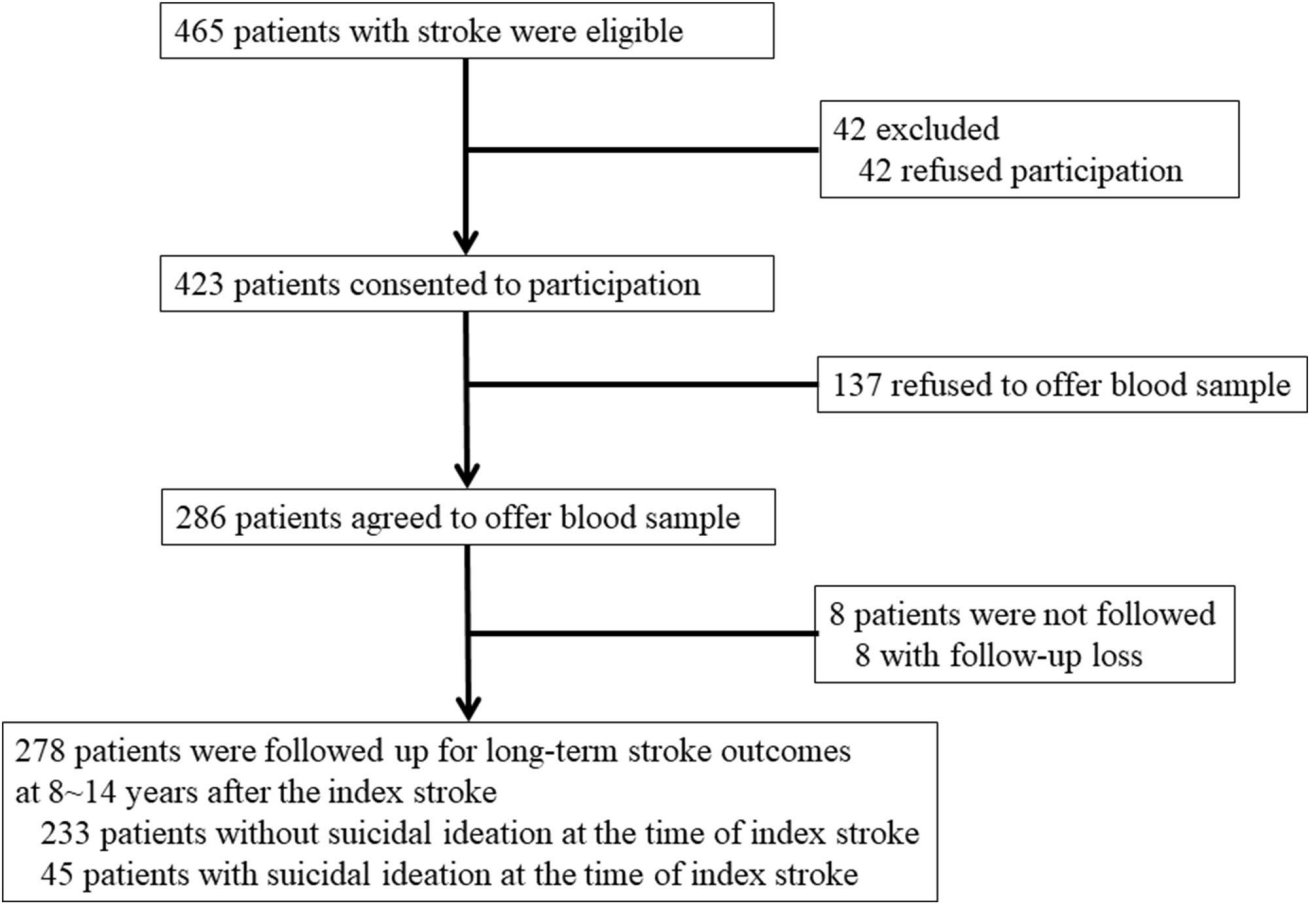
Study outline and participant recruitment process.

All analyses were performed using data collected prospectively for a naturalistic investigation examining psychiatric disorders in stroke survivors^25^. The recruitment process is outlined in Fig. 1. Participants were consecutively recruited from among patients with recent ischemic stroke (N = 465) referred to the Department of Neurology, Chonnam National University Hospital (CNUH), Gwangju, South Korea, between 2005 and 2011. The patients were managed by neurologists according to published guidelines^26^. Patients who met the eligibility criteria of this study (detailed in Supplementary Materials) and consented to participate (N = 423) were evaluated as inpatients. Data on clinical characteristics, including SI in the acute phase (2 weeks after stroke), were obtained. In total, 286 (67.6%) patients agreed to blood sampling for genetic tests. All participants were approached for follow-up evaluations of cerebro-cardiovascular outcomes in 2019, 8–14 years after the index stroke. The 278 patients (97.2%) who were followed up comprised the baseline sample for the present analyses.

The study was approved by the CNUH Institutional Review Board and conducted in accordance with the 1964 Helsinki declaration and its later amendments or comparable ethical standards. Written informed consent was obtained from each participant after providing both written and oral information prior to study entry.

### Acute phase evaluations of suicidal ideation and clinical covariates

The presence of SI was determined using the “suicidal thoughts” item of the Montgomery–Åsberg Depression Rating Scale (MADRS)^27^, which was administered by two research nurses blinded to the M.I.N.I. results and trained and supervised by the project psychiatrist. This item assesses the extent to which respondents feel that life is not worth living, and whether they have plans to commit suicide. Scores range between 1 (satisfied with life) and 6 (explicit plans for suicide). A score of 2 (fleeting suicidal thoughts) or higher on this item was taken to indicate the presence of SI, consistent with a previous study of SI after stroke^28^.

A variety of characteristics potentially associated with SI in stroke patients^18^ were included as covariates in the present analyses. Data on sociodemographic and clinical characteristics, including age, gender, years of education, marital status, employment status, previous history of stroke, and presence of cardiac disease, were obtained from patients and their caregivers. Stroke severity was measured using the National Institutes of Health Stroke Scale (NIHSS)^29^, and stroke location was categorized as left, right, or bilateral hemisphere, and further subdivided as anterior, posterior, or both based on brain magnetic resonance imaging (MRI) scans. Due to the close correlation between SI and poststroke depression (PSD)^30^, PSD was assessed using the Mini International Neuropsychiatric Interview (M.I.N.I.)^31^, a structured diagnostic psychiatric interview based on criteria of the Diagnostic and Statistical Manual of Mental Disorders, 4th edition (DSM-IV). The M.I.N.I. ver. 5.0.0, which was formally translated and standardized in Korean^32^, was administered by experienced study psychiatrists.

### *SLC6A4* gene methylation analysis

Venous blood samples (5 ml) were obtained from stroke patients who agreed to participate in genetic testing. Genomic DNA (1 µg) was extracted from whole blood (200 μl) using the QIAamp DNA Blood Mini Kit (Qiagen; Valencia, CA, USA) in the laboratory of Chonnam National University Hwasun Hospital; analyses were completed by colleagues who were blind to the clinical data. DNA methylation status only in the promotor region of SLC6A4 gene was determined using the PSQ 96 M Pyrosequencing System (Biotage AB, Uppsala, Sweden), as described previously^33,34^. The *SLC6A4* promoter region for analyzing methylation status (Supplementary Fig. 1) has been deposited in GenBank (accession number: BankIt1577778 SLC6A4 KC106430). A CpG-rich region of the promoter between – 479 and – 350, relative to the transcriptional start site, including seven CpG sites, was analyzed, as has been the case in other studies of psychiatric symptoms in a general population^13,33^ as well as in stroke patients^34^. More details of the methods and sites investigated are provided in the Supplementary Materials and Supplementary Fig. 1. Percentage data for methylation at seven CpG sites, and the average value, were utilized in the analyses.

### Long-term stroke outcomes

Comprehensive evaluations of long-term stroke outcomes, including recurrent stroke, myocardial infarction, and vascular death, were assessed during the follow-up period. Recurrent stroke was defined using the same criteria applied to the index stroke event. Myocardial infarction was defined by the presence of at least two of the following: symptoms of myocardial ischemia, changes in cardiac enzymes, and electrocardiography indicative of myocardial infarction^35^. Vascular death was defined as death due to recurrent stroke, myocardial infarction, or heart failure, or sudden death without an identifiable nonvascular cause, as in a previous publication^36^.

All participants were followed up, with electronic medical records checked regarding the above outcomes. Patients were asked whether they had ever visited a hospital for the management of stroke or myocardial infarction. Information on deceased patients was obtained from caregivers or physicians using structured questionnaires; death certificates were also obtained. All patients were followed up to the present time, or until death. Due to small numbers of cases with a single CCVE, the primary endpoint in this study was composite CCVEs defined by sum of recurrent stroke, myocardial infarction, and vascular death (Supplementary Table S1). The secondary endpoint was individual events, including recurrent stroke, myocardial infarction, or vascular death. An independent committee composed of neurologists blinded to the participants’ depression status assessed all CCVEs.

### Statistical analyses

Seven CpG sites on the *SLC6A4* gene and their average value were classified as low or high methylation using the median value (Supplementary Table S2), similar to a previous investigation^37^. Baseline demographic and clinical characteristics were analyzed based on methylation and SI status using t-tests, χ2 tests, or Fisher’s exact test as appropriate. Characteristics significantly associated with methylation and SI status (P < 0.05), and other variables with potential effects on long-term stroke outcomes^38,39^, were used as covariates in further analyses. Associations of the methylation status of individual CpG sites (and the average *SLC6A4* methylation value), and of SI immediately after stroke (within 2 weeks), with the occurrence of individual and composite CCVEs were determined using Cox proportional hazards models with and without adjustment for covariates. The interactive effect of *SLC6A4* methylation status and SI on composite or individual CCVEs was also computed in the Cox proportional hazards models, for the total population and the population stratified by SI. All statistical tests were two-sided, with the significance level set at 0.05. Statistical analyses were carried out using SPSS software (ver. 21.0; SPSS Inc., Chicago, IL, USA).

## Results

### *SLC6A4* methylation status and baseline characteristics

In terms of clinical characteristics and long-term stroke outcomes, no significant differences were found between the participants who consented to blood sampling and those who did not (all *p*-values > 0.1). Of the 278 patients who were followed up, 45 (16.2%) experienced SI immediately (within 2 weeks) after stroke. Participants with SI were more likely to have a diagnosis of depression based on the DSM-IV criteria, and to experience more severe disability just after the index stroke (as measured by the NIHSS; Supplementary Table S3).

The median (interquartile range) and mean (standard deviation) *SLC6A4* methylation percentage values of seven individual CpG sites and the average value, are provided in Supplementary Table S2. Due to the close correlations between the methylation values for the individual CpG sites and the average value (all Spearman’s rho > 0.045, *p* ≤ 0.001), the average value was the primary outcome. There was no significant difference in any clinical characteristic by methylation status (low vs. high; all *p*-values > 0.05; Supplementary Table S4).

### Associations of *SLC6A4* methylation status and suicidal ideation with long-term stroke outcomes

All participants were followed for 8–14 years until 2019, or until they died (median follow-up 12.0 [interquartile range, 9.8–13.1 years; mean, 10.3 [SD, 4.1] years). Composite CCVEs occurred in 76 participants (27.3%). Long-term stroke outcomes (individual or composite CCVEs) by SI and *SLC6A4* methylation status are displayed in Tables 1 and 2, calculated using Cox proportional hazards models. Stroke patients with SI at 2 weeks were more likely to experience composite CCVEs, recurrent stroke and vascular death during the 8–14-year follow-up period, according to unadjusted analyses. SI at 2 weeks after stroke was significantly associated with composite CCVEs and recurrent stroke only after adjusting for age, previous stroke, NIHSS score, the presence of cardiac disease, and depression (according to the DSM-IV criteria). Patients with a higher average *SLC6A* gene methylation value were more likely to suffer from composite CCVEs, recurrent stroke, and myocardial infarction in unadjusted analyses, but the association with myocardial infarction disappeared after adjustment for covariates.

**Table 1:** Association of suicidal ideation with long-term stroke outcomes (cumulative incidence, %).

Event	Suicidal ideation status	Number of patients	Events, N (%)	Unadjusted HR (95% CI)	Adjusted^a^	
					HR (95% CI)	*p*-value
Composite CCVEs	Absence	233	54 (23.2)	Ref	Ref	0.015
	Presence	45	22 (48.9)	2.50 (1.52–4.11)	2.12 (1.16–3.88)	
Recurrent stroke	Absence	233	35 (15.0)	Ref	Ref	0.002
	Presence	45	17 (37.8)	2.94 (1.65–5.26)	3.25 (1.57–6.76)	
Myocardial infarction	Absence	233	14 (6.0)	Ref	Ref	0.434
	Presence	45	2 (4.4)	0.81 (0.18–3.55)	0.53 (0.11–2.61)	
Vascular death	Absence	233	12 (5.2)	Ref	Ref	0.090
	Presence	45	7 (15.6)	3.30 (1.30–8.39)	2.72 (0.85–8.64)	

HR (95% CI) were calculated using Cox proportional hazards models.

^a^Adjusted for age, NIHSS score, previous history of stroke, presence of cardiac disease and DSM-IV depression at 2 weeks after stroke.

HR, Hazard ratios; CI, confidence interval; CCVEs; Cerebro-cardiovascular events, NIHSS, National Institutes of Health Stroke Scale; DSM-IV, Diagnostic and Statistical Manual of Mental Disorders, 4th edition.

**Table 2:** Association of the average *SLC6A4* methylation value with long-term stroke outcomes (cumulative incidence, %) in the overall cohort, and stratified by suicidal ideation status.

Event	Patients group	Methylation type	Patient numbers	Events, N (%)	Unadjusted HR (95% CI)	Adjusted^a^		*p*-value for interaction^b^
						HR (95% CI)	*p*-value	
Composite	All patients	Lower	138	25 (18.1)	Ref	Ref		
		Higher	140	51 (36.4)	2.32 (1.43–3.74)	2.19 (1.34–3.56)	0.002	
	**SI**							
	Absence	Lower	120	21 (17.5)	Ref	Ref		0.113
		Higher	113	33 (29.2)	1.82 (1.05–3.15)	1.77 (1.02–3.07)	0.044	
	Presence	Lower	18	4 (22.2)	Ref	Ref		
		Higher	27	18 (66.7)	4.45 (1.49–13.31)	4.82 (1.40–16.60)	0.013	
Recurrent stroke	All patients	Lower	138	19 (13.8)	Ref	Ref		
		Higher	140	33 (23.6)	1.92 (1.09–3.37)	1.91 (1.08–3.40)	0.027	
	**SI**	Lower						
	Absence	Higher	120	15 (12.5)	Ref	Ref		0.248
		Lower	113	20 (17.7)	1.50 (0.77–2.93)	1.56 (0.79–3.08)	0.200	
	Presence	Higher	18	4 (22.2)	Ref	Ref		
		Lower	27	13 (72.2)	3.16 (1.04–9.87)	3.85 (1.02–14.48)	0.046	
Myocardial infarction	All patients	Lower	138	4 (2.9)	Ref	Ref		
		Higher	140	12 (8.6)	3.10 (1.00–9.60)	2.59 (0.83–8.10)	0.103	
	**SI**							
	Absence	Lower	120	4 (3.3)	Ref	Ref		NA
		Higher	113	10 (8.8)	2.68 (0.84–8.56)	2.25 (0.69–7.32)	0.179	
	Presence	Lower	18	0 (0)	Ref	Ref		
		Higher	27	2 (7.4)	49.9 (0–5,279,309.2)	201,736.6 (NA)	0.979	
Vascular death	All patients	Lower	138	6 (4.3)	Ref	Ref		
		Higher	140	13 (9.3)	2.24 (0.85–5.91)	1.98 (0.75–5.27)	0.170	
	**SI**							
	Absence	Lower	120	6 (5.0)	Ref	Ref		NA
		Higher	113	6 (5.3)	1.09 (0.35–3.37)	0.96 (0.31–3.02)	0.948	
	Presence	Lower	18	0 (0)	Ref	Ref		
		Higher	27	7 (25.9)	50.8 (0.11–24,364.3)	388,932.3 (0–9.51e + 237)	0.962	

The higher and lower methylation were classified using the median value.

HR (95% CI) were calculated using Cox proportional hazards models.

^a^Adjusted for age, NIHSS score, previous history of stroke, presence of cardiac disease and DSM-IV depression at 2 weeks after stroke.

^b^The interactive effect between average *SLC6A4* methylation value and suicidal ideation on CCVEs were calculated in the same adjusted model.

HR, Hazard ratios; CI, confidence interval; CCVEs; cerebro-cardiovascular events, SI, suicidal ideation; NIHSS, National Institutes of Health Stroke Scale; DSM-IV, Diagnostic and Statistical Manual of Mental Disorders, 4th edition.

### Individual and interactive effects of the average *SLC6A4* methylation value and suicidal ideation on long-term stroke outcomes

**Figure 2:**
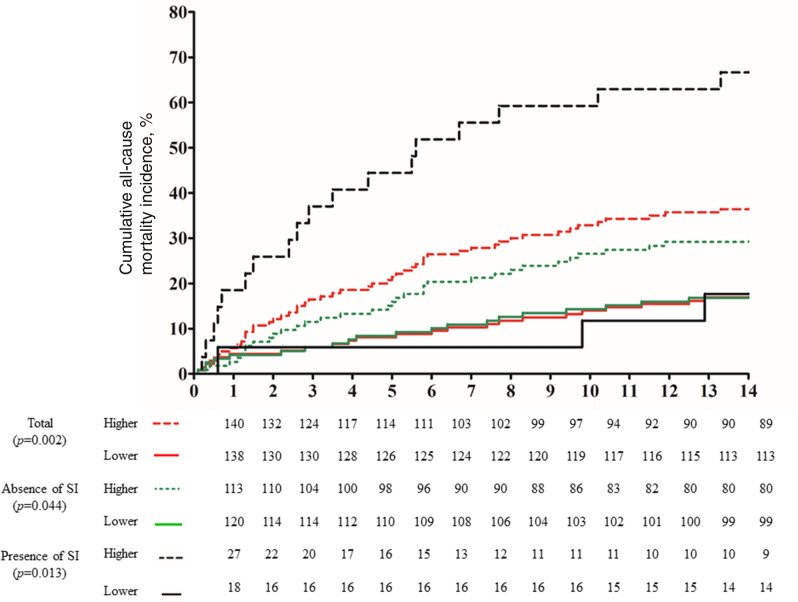
Association of the average *SLC6A4* methylation value with the cumulative incidence (%) of composite cerebro-cardiovascular events, stratified by SI status immediately after stroke (within 2 weeks). Cox proportional hazards models were used for analyses of the overall cohort, and for analyses stratified by SI after adjustment for age, NIHSS score, previous history of stroke, presence of cardiac disease, and depression (according to the DSM-IV criteria) within 2 weeks after stroke. The interaction effect between average SLC6A4 methylation value and SI on composite CCVEs was not significant (*p* = 0.113). Abbreviations: SI, suicidal ideation; NIHSS, National Institutes of Health Stroke Scale; DSM-IV, Diagnostic and Statistical Manual of Mental Disorders, 4th edition.

Interactive effects of SI and the average *SLC6A4* methylation value on composite and individual CCVEs are summarized in Table 2 and presented visually in Fig. 2. The association between the average *SLC6A4* methylation value and composite CCVEs was significant, especially in patients with SI at 2 weeks after stroke. However, the interaction effect between the average *SLC6A4* methylation value and SI status on composite CCVEs was not statistically significant. A higher average *SLC6A4* methylation value was a significant predictor of recurrent stroke only in the presence of SI at 2 weeks after stroke, but did not predict any other CCVEs.

### Individual and interactive effect of the methylation status of individual CpG sites and suicidal ideation on long-term stroke outcomes

**Figure 3:**
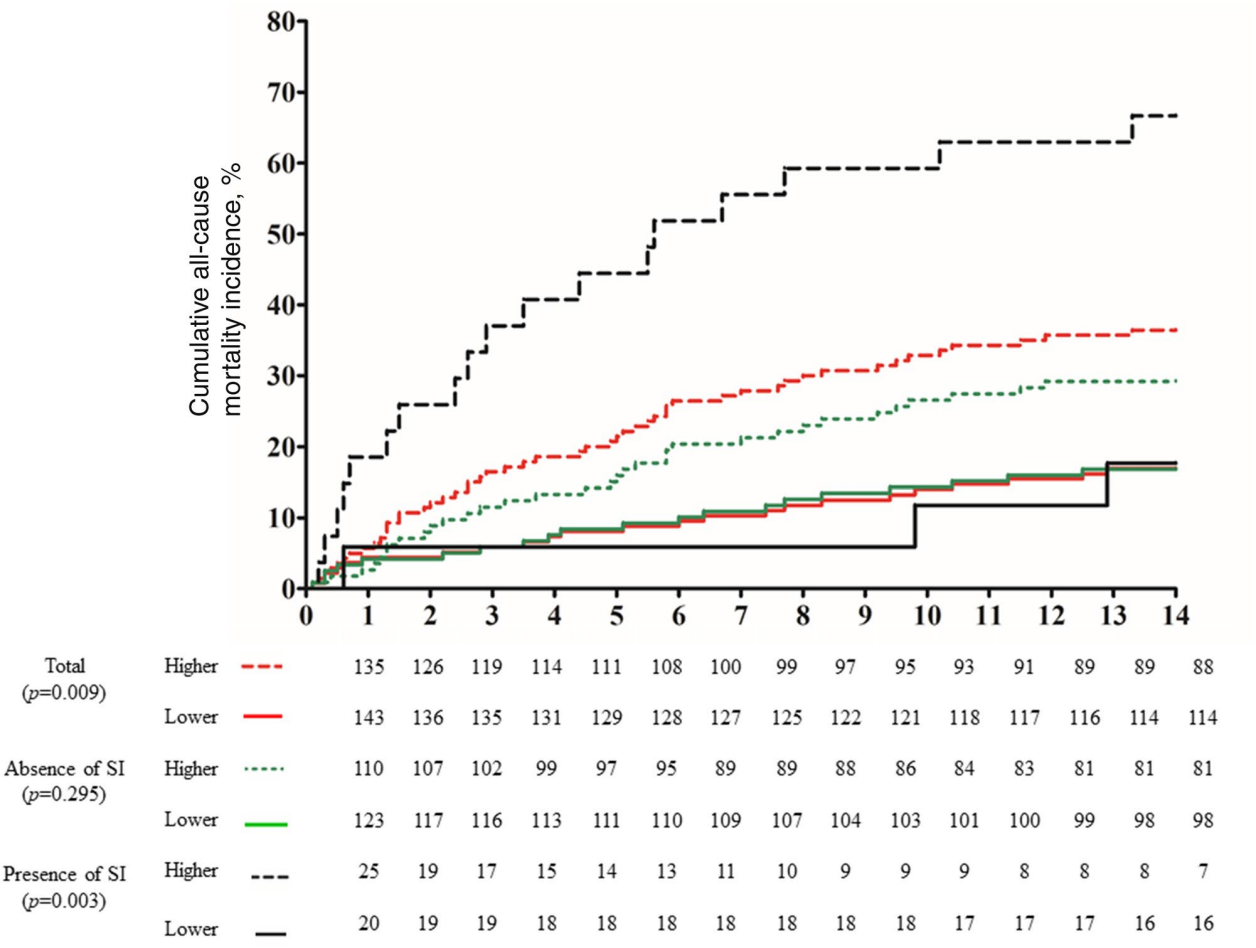
Association of the methylation status of CpG 4 with the cumulative incidence (%) of composite cerebro-cardiovascular events, stratified by SI status immediately after stroke (within 2 weeks). Cox proportional hazards models were used for analyses of the overall cohort, and for analyses stratified by SI after adjustment for age, NIHSS score, previous history of stroke, presence of cardiac disease, and depression (according to the DSM-IV criteria) within 2 weeks after stroke. The interaction effect between methylation status of CpG 4 and SI on composite CCVEs was significant (*p* = 0.012).

The associations of the methylation status of individual CpG sites on long-term stroke outcomes (individual and composite CCVEs), and their interactive effect with SI, are summarized in Supplementary Tables S5–S11. There was a tendency for stroke patients with CpG sites having a high methylation status to experience more composite CCVEs (Fig. 3 and Supplementary Figure S2), especially in patients who experienced SI at 2 weeks after stroke. The methylation status of CpGs 1, 2, 4, and 6 was significantly associated with composite CCVEs in the patients with SI; this was only the case for CpG 2 in patients without SI. Only the methylation status of CpG 4 and SI showed an interaction effect on composite CCVEs (*p* = 0.012).

In terms with other CCVEs, a high methylation status for CpGs 1 and 4 was significantly associated with recurrent stroke in the overall cohort, and in the presence (but not the absence) of SI in stratified analyses. However, only the methylation status of CpG 4 and SI showed an interaction effect on recurrent stroke (*p* = 0.039). A high methylation status for CpG 2 was significantly associated with recurrent stroke in the overall cohort, and CpG 6 hypermethylation was significantly associated with recurrent stroke in both the overall cohort and in patients without (but not with) SI in stratified analyses. Only the methylation status of CpG 6 and SI showed an interaction effect on vascular death (*p* = 0.038).

## Discussion

The principal findings of this study were that both high average *SLC6A4* methylation values and SI at 2 weeks predicted worse long-term stroke outcomes, i.e., composite CCVEs and recurrent stroke, at 8–14 years after stroke, independent of covariates. The associations of the methylation status of CpGs 1, 2, and 4, and the average *SLC6A4* methylation value, with long-term stroke outcomes (composite CCVEs and recurrent stroke) were stronger in patients who experienced SI within 2 weeks of stroke; a significant interaction effect of high CpG 4 methylation status and SI on long-term stroke outcomes was also seen.

High methylation status for CpGs 1, 2, 4, and 6 was associated with composite CCVEs, and high methylation status of CpGs 1 and 4 was associated with recurrent stroke. Plausible mechanisms for the association between *SLC6A4* hypermethylation and poor long-term stroke outcomes is as follows. The serotonin transporter is a key regulator of 5-HT levels, and its function is affected by epigenetic mechanisms including methylation of the *SLC6A4* gene^21,40^. Hypermethylation of the *SLC6A4* gene was associated with decreased *SLC6A4* mRNA^13,21^, gene activity^40^, brain 5-HT synthesis^21^, and availiability^41^. Stroke patients with *SLC6A4* hypermethylation might have reduced 5-HTT function and 5-HT availability, which would lead to decreased BDNF expression and thus suppress neurogenesis^42,43^ and promote inflammation^44^. This, in turn, could result in dysregulating cerebro-cardiovascular functions through platelet aggregation in both the brain and periphery. These mechanisms might hamper stroke recovery and increase the likelihood of CCVEs^5^.

In this study, SI within 2 weeks after stroke predicted worse long-term stroke outcomes. To our knowledge, this is the first study to report this finding. Although the suicide rate in stroke patients is approximately double that in the general population^30,45^, and SI is often a precursor to actual suicidal behavior, the utility of SI for predicting long-term stroke outcomes has not been investigated. SI is one of the diagnostic criteria for major depressive disorders^46^ and is obviously strongly associated with depression^18^. The association between SI within 2 weeks after stroke and long-term CCVEs in this study remained significant even after adjustment for depression. This association might be mediated by the biological mechanisms underlying suicidality, including neuroendocrine dysfunction, enhanced inflammatory and autonomic responses, and reduced neuroplasticity^47^, which are also associated with poorer cerebro-cardiovascular function^48–50^. With regard to behavioral mechanisms, stroke patients with SI might have difficulty coping with the stress associated with the stroke, and maintaining healthy behaviors including regular exercise, hospital visits, and medication use, all of which have a role in stroke outcomes. Our findings suggest that SI has effects beyond suicidal behavior, including on long-term cerebro-cardiovascular outcomes, in patients experiencing stroke, which indicates the necessity of careful evaluation and appropriate management of stroke patients showing SI, particularly at 2 weeks after stroke^30,51^.

Hypermethylation of CpGs 1 and 4, and a high average *SLC6A4* methylation value, predicted poor long-term stroke outcomes (composite CCVEs and recurrent stroke), especially in patients who experienced SI within 2 weeks of stroke. Our findings suggest that methylation and SI might have synergistic effects on long-term stroke outcomes. Previously, suicidality was shown to be associated with low 5-HT^22^, and *SLC6A4* hypermethylation would exacerbate any deficit in 5HT^21,41^. Furthermore, post-stroke depression (PSD) is a well-known risk factor for poor long-term stroke outcomes^52^, and SI after stroke is strongly associated with PSD^30,45^ and *SLC6A4* hypermethylation has been proposed as a diagnostic and prognostic biomarker for PSD^34^. These two risk factors (SI and *SLC6A4* hypermethylation) contributed to increase PSD risk which mediate the worse long-term stroke outcomes additively.

For association with individual CpG sites in the present analyses, previous studies found that low methylation status for CpGs 1, 4, and 6 was related to depression in pregnancy^53^. On the other hand, high methylation status for CpG 2 was associated with higher perceived stress while hypermethylation of CpGs 1, 2, and 4 was associated with more severe depression^33^. In previous studies of stroke, hypermethylation of CpGs 1, 4, and 5 was associated with PSD at 2 weeks, and hypermethylation of CpGs 3, 5, 6, and 7 was associated with PSD at 1 year^34^. Although there may be differential associations of the methylation status of various CpGs with psychiatric symptoms, further investigations are needed to determine the precise mechanisms, including in relation to 5-HT expression.

Before drawing conclusions, several methodological issues of this study should be considered. First, the “suicidal thoughts” MADRS item was used to ascertain the presence of SI, rather than a dedicated instrument. However, the validity of suicide-related MADRS items has been proven in previous studies of suicide^54^, and this approach was used in a randomized controlled trial^55^. Also, SI, but not suicide attempts or completed suicides, was investigated in our study. Although SI is closely related to suicidal behavior^56^, it is difficult to generalize our findings to overall suicidal behavior in stroke patients. Second, our stroke patients were recruited from a single center, which may also limit the generalizability of the findings, albeit that single-center studies benefit from consistency in evaluation and treatment. Third, drug treatment, including antidepressants, during follow-up was not considered in our analyses due to a lack of data, although this is a factor that affects both methylation status and long-term stroke outcomes. Additionally, the methylation status was only measured only once, at the time of the index stroke. Thus, the association between methylation changes during the follow-up period and long-term stroke outcomes remains uncertain. Fourth, the functional relevance of *SLC6A4* hypermethylation, including the expression level of 5-HTT mRNA and the serotonin level in both the periphery and the brain, was not explored. Furthermore, methylation status could be tissue specific, and *SLC6A4* methylation in the present study was measured in the peripheral blood but not in the brain. However, the *SLC6A4* hypermethylation status in the periphery affected low transcriptional activity of 5-HTT mRNA^21^ and was associated with decreased brain 5-HT synthesis and availability^21,41^. Also, a previous investigation of nine CpGs which included all of CpG sites in the present analyses, revealed that increased methylation of the investigated region conferred decreased gene activity^40^. Thus, future research will be needed to understand the functional relevance of *SLC6A4* hypermethylation and the association of methylation status between peripheral blood and brain tissue. Finally, the sample size was modest, and there were insufficient numbers of CCVEs during the 8–14-year follow-up to detect statistical differences among them. Thus, caution is warranted when interpreting the associations between the methylation status of individual CpG sites and long-term outcomes, such as myocardial infarction and vascular death. Our study also had several strengths. All eligible patients recently suffering a stroke were recruited consecutively, which increased sample homogeneity. Also, a range of psychiatric and stroke-related covariates, assessed using a well-validated scale, were included in the analyses.

In conclusion, stroke patients with a high SLC6A4 gene methylation value, and/or with SI within 2 weeks after stroke, were more likely to experience composite CCVEs and recurrent stroke. The utility of *SLC6A4* hypermethylation for predicting long-term stroke outcomes was superior in patients who experienced SI within 2 weeks of stroke. Stroke patients more likely to experience poor outcomes can be identified through tests of methylation status and evaluation of SI. Such evaluations could improve long-term outcomes in high-risk stroke patients, in conjunction with intensive treatment.

## Supplementary Information

Supplementary Information

## Abbreviations

SI: Suicidal ideation
NIHSS: National Institutes of Health Stroke Scale
DSM-IV: Diagnostic and Statistical Manual of Mental Disorders, 4th edition
