# Supplementary material for: Association of SLC6A4 methylation with long-term outcomes after stroke: focus on the interaction with suicidal ideation
Source: Sci Rep. 2021 Feb 1;11:2710. doi: 10.1038/s41598-021-81854-9 (PMC7851135; doi:10.1038/s41598-021-81854-9)
Supplement: Supplementary file 1 — Supplementary Information [file 41598_2021_81854_MOESM1_ESM.docx]

**Association of *SLC6A4* methylation with long-term outcomes after stroke: Focus on the interaction with suicidal ideation**

Hee-Ju Kang^1^, Eun-Hye Lee^1^, Ju-Wan Kim^1^, Sung-Wan Kim^1^, Il-Seon Shin^1^, Joon-Tae Kim^2^, Man-Seok Park^2^, Ki-Hyun Cho^2^, Jung-Soo Han^3^, In Kyoon Lyoo^4^, Jae-Min Kim^1*^

***Supplemental information***

**SUPPLEMENTAL METHODS……………………………………………………………..2**

Eligible criteria ………….. ………………………..………………..………………………..2

*SLC6A4* DNA methylation analysis.. ……………..………………..………………………...2

**SUPPLEMENTAL FIGURE S1….…………..……………………………………………..4**

**SUPPLEMENTAL FIGURE S2….…………..……………………………………………..5**

**SUPPLEMENTAL Table S1.………………….………………………….………………...9**

**SUPPLEMENTAL Table S2.………………….………………………….………………...10**

**SUPPLEMENTAL Table S3.………………….………………………….………………...11**

**SUPPLEMENTAL Table S4.………………….………………………….………………...12**

**SUPPLEMENTAL Table S5.………………….………………………….………………...13**

**SUPPLEMENTAL Table S6.………………….………………………….………………...15**

**SUPPLEMENTAL Table S7.………………….………………………….………………...17**

**SUPPLEMENTAL Table S8.………………….………………………….………………...19**

**SUPPLEMENTAL Table S9.………………….………………………….………………...21**

**SUPPLEMENTAL Table S10………………….………………………….………………..23**

**SUPPLEMENTAL Table S11.………………….………………………….………………..25**

**SUPPLEMENTAL METHODS**

**Eligible criteria**

All patients who were hospitalized for acute stroke at the study site and who were eligible to participate were recruited for the present study. The inclusion criteria were as follows: i) ischemic stroke diagnosed by brain magnetic resonance imaging (MRI); ii) the capacity to complete the entire investigation and all questionnaires; and iii) the ability to comprehend the study objectives and provide informed consent. The exclusion criteria were as follows: i) severe physical conditions that were life threatening or hampered stroke recovery; ii) disturbances in communication due to dysphasia or dysarthria that prevented the provision of informed consent and/or completion of the questionnaires; iii) other comorbid neurological or psychiatric conditions including dementia, Parkinson’s disease, brain tumor, epilepsy, schizophrenia and other psychotic disorder including schizoaffective, schizophreniform and delusional disorders and/or alcohol and substance dependence; iv) severe medical diseases resulting in disturbances of movement prior to stroke; and v) a Mini-Mental State Examination (MMSE)^1^ score <16 at the baseline evaluation.

***SLC6A4* DNA methylation analysis**

Genomic DNA (1 µg) was extracted from leukocytes with the QIAamp DNA Blood Mini Kit (Qiagen; Valencia, CA, USA) and then treated with bisulfite using the EpiTech Bisulfite Kit (Qiagen) according the manufacturer’s protocol. A 130bp fragment of *SLC6A4* promoter was amplified by PCR from bisulfite-treated DNA using the forward and reverse primers designated in Supplementary Figure 1. PCR conditions were 95ºC for 15 minutes, followed by 45 cycles of 95 ºC for 15 seconds, 57 ºC for 30 seconds, and 72 ºC for 15 seconds, with a final extension of 5 minutes at 72ºC. PCR products were sequenced using the PSQ 96M Pyrosequencing system (Biotage) according to the manufacturer’s protocol with the following sequencing primers designated in Supplementary Figure 1. The methylation percentage at each CpG region was quantified using the Pyro Q-CpG software, version 1.0.9 (Biotage).

**Reference of supplemental materials**

1. Folstein, M.F., Fostein, S.E. & McHugh, P.R. “Mini-Mental State” a practical method for grading the cognitive state of patients for the clinician. *J. Psychiatr. Res.* **12,** 189-198 (1975).

**SUPPLEMENTAL FIGURE S1 *SLC6A4* promoter region analyzed in terms of methylation status**


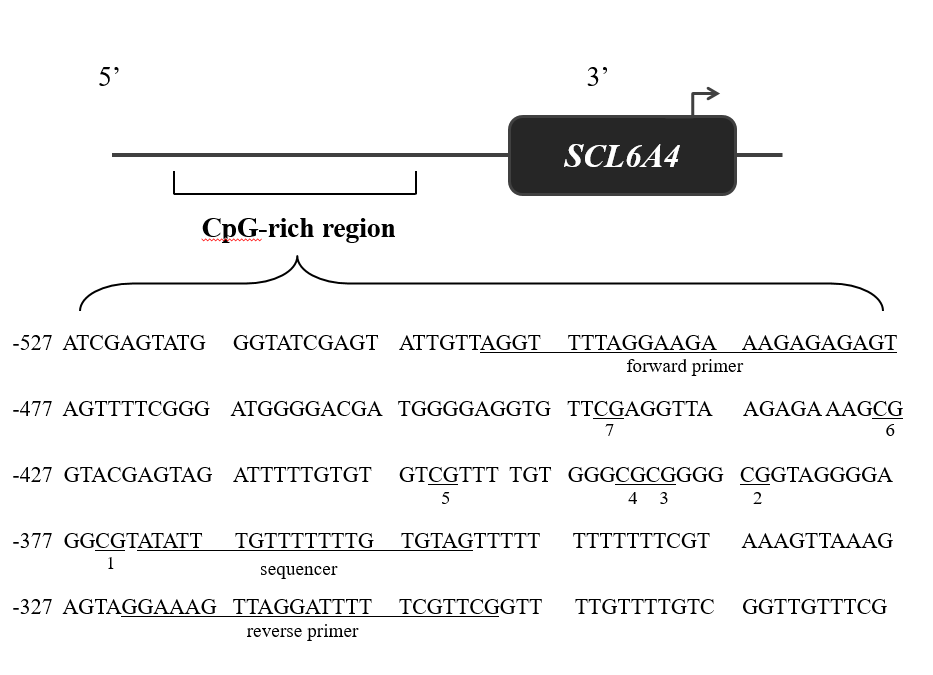


**Figure legends**

The CpGs are underlined and numbered. Forward and backward primers and sequencer are designated. Numbering of the gene sequence is relative to the transcriptional start site.

**SUPPLEMENTAL FIGURE S2** **Associations of the methylation status of individual CpGs with the cumulative incidence (%) of composite cerebro-cardiovascular events (CCVEs; cumulative incidence, %), stratified by suicidal ideation status immediately after stroke (within 2 weeks).**

**Figure legend:** Cox proportional hazards models were used for analyses of the overall cohort, and for analyses stratified by SI after adjustment for age, NIHSS score, previous history of stroke, presence of cardiac disease, and depression (according to the DSM-IV criteria) within 2 weeks after stroke.

Abbreviations: SI, suicidal ideation; NIHSS, National Institutes of Health Stroke Scale; DSM-IV, Diagnostic and Statistical Manual of Mental Disorders, 4^th^ edition

1) CpG 1: The interaction effect between methylation status and SI on composite CCVEs was at borderline significance (*p* =0.066)


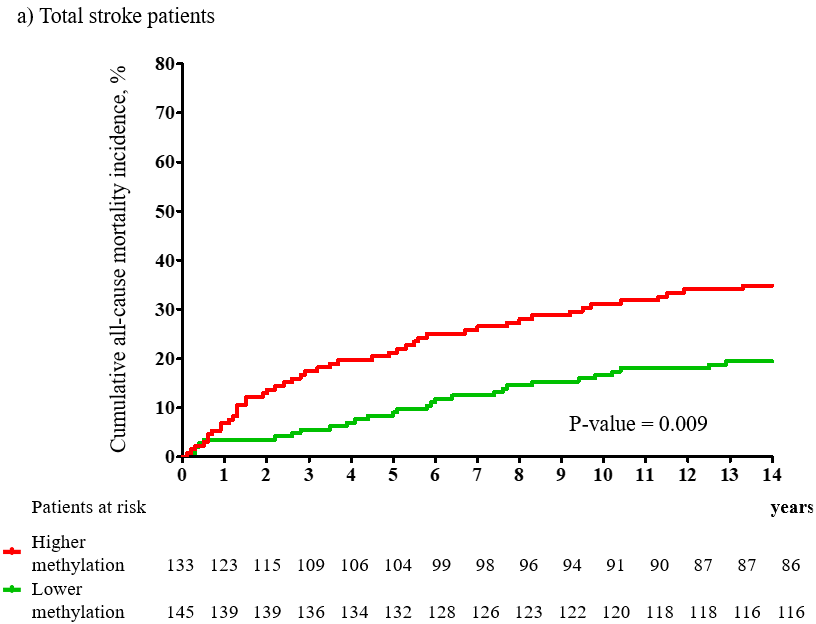

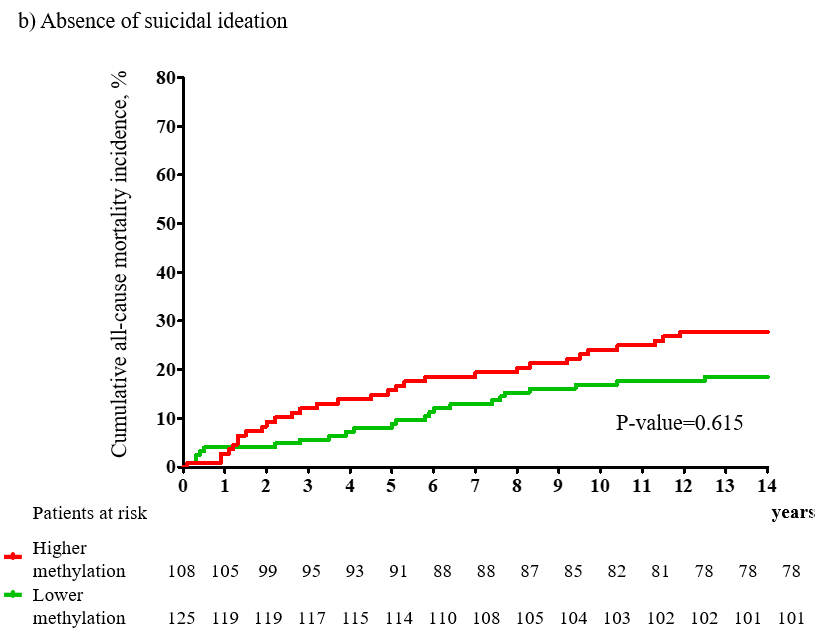

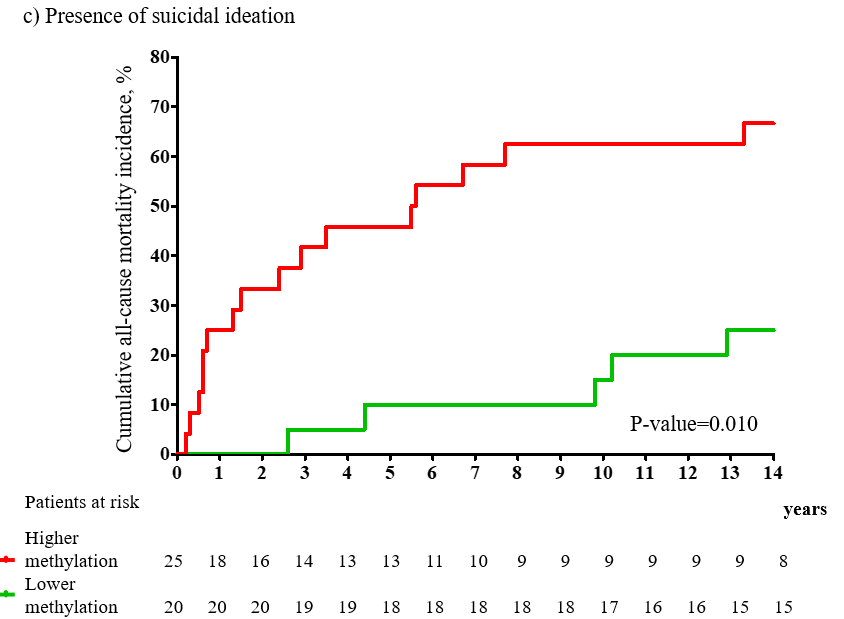


2) CpG 2: The interaction effect between methylation status and SI on composite CCVEs was not significant (*p* =0.552)


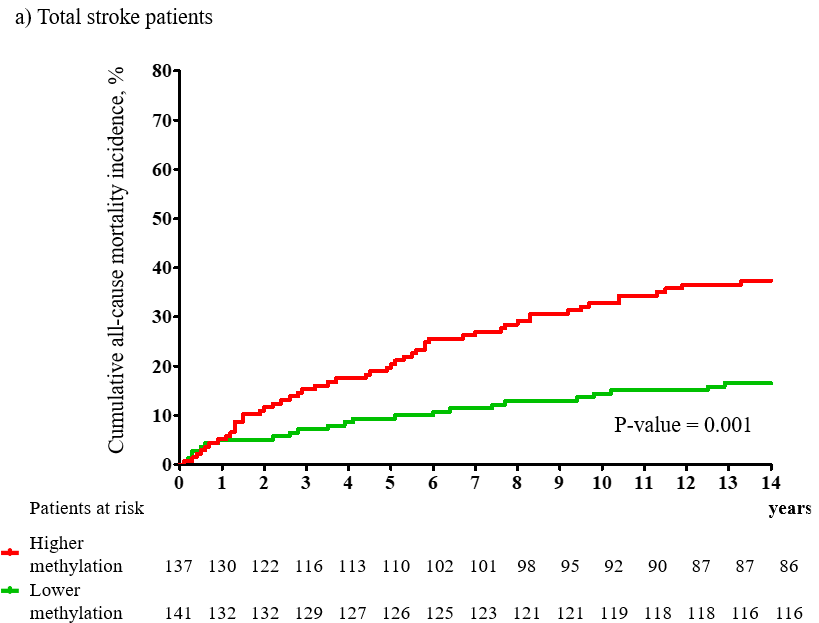

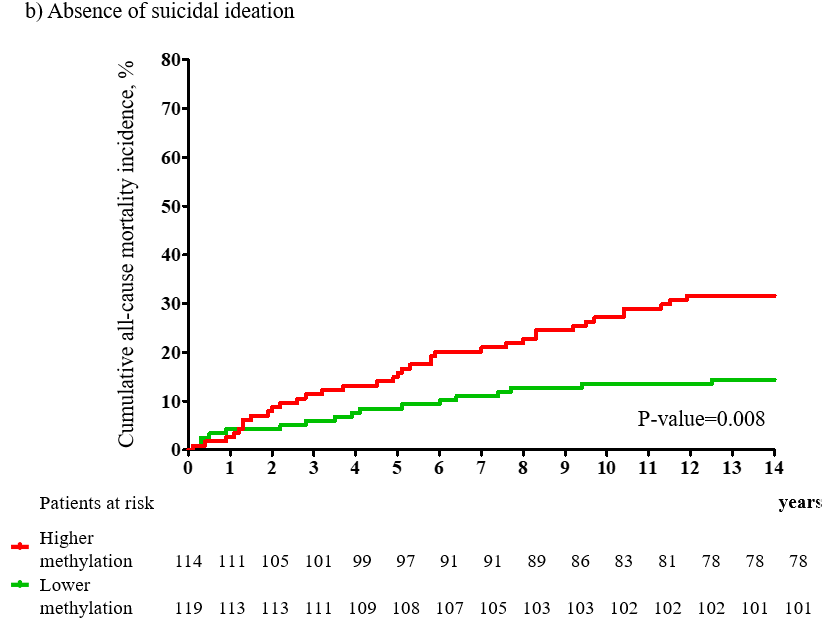

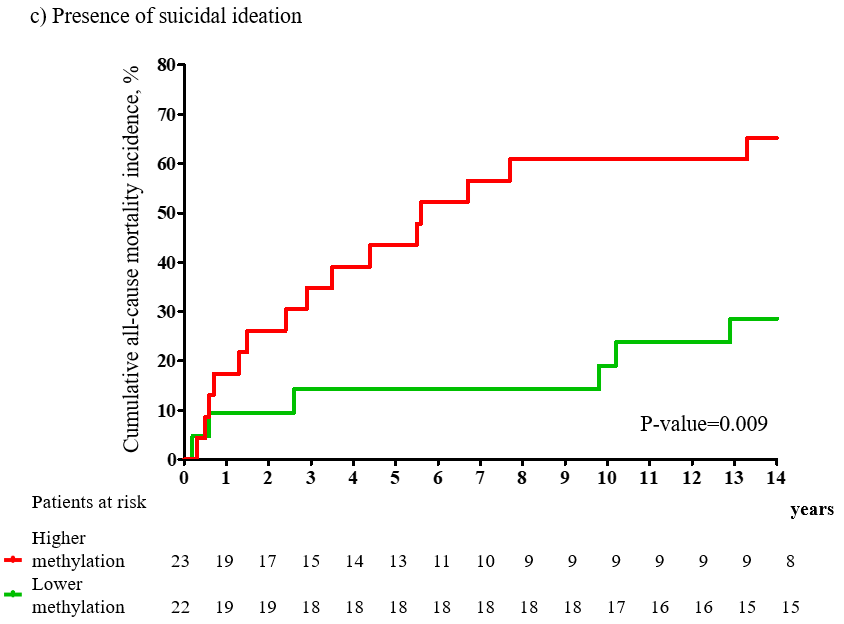


3) CpG 3: The interaction effect between methylation status and SI on composite CCVEs was not significant (*p* =0.362)


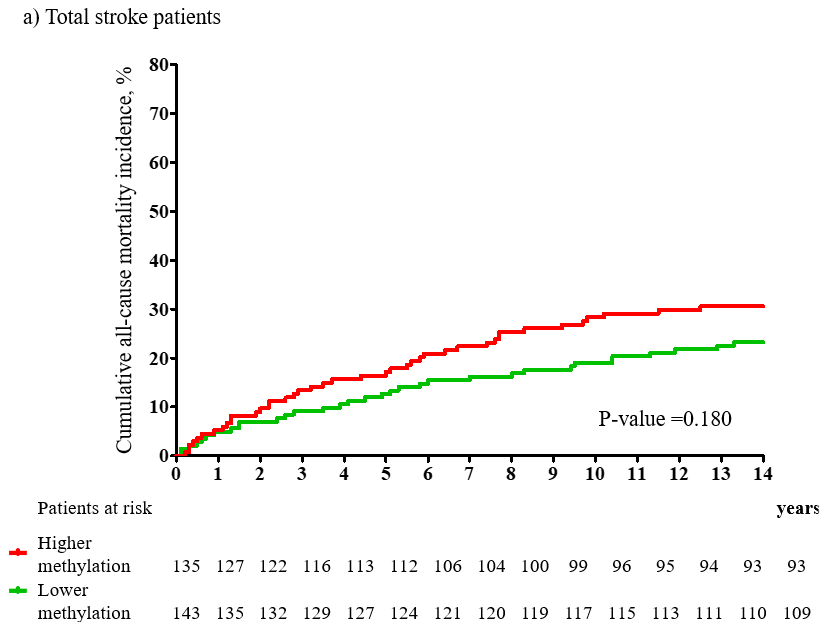

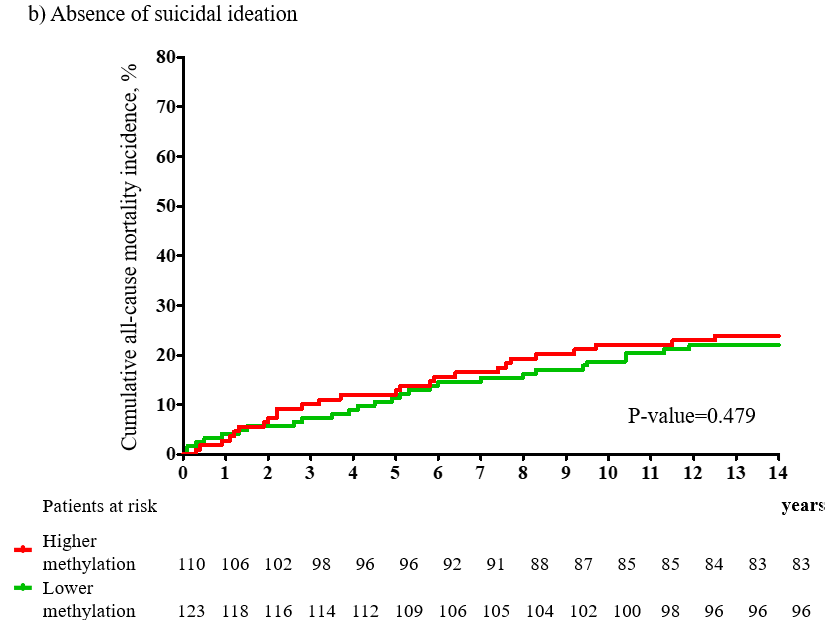

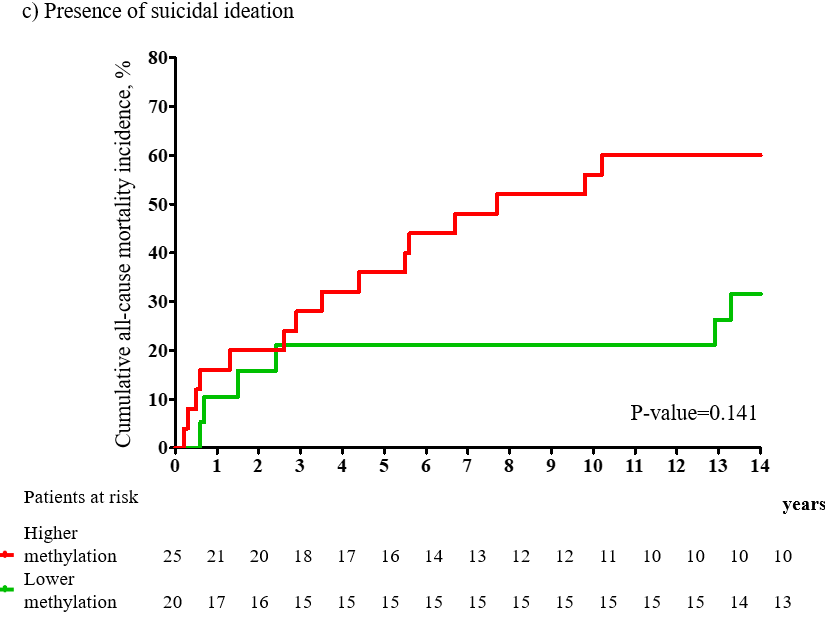


4) CpG 5: The interaction effect between methylation status and SI on composite CCVEs was not significant (*p* =0.586)


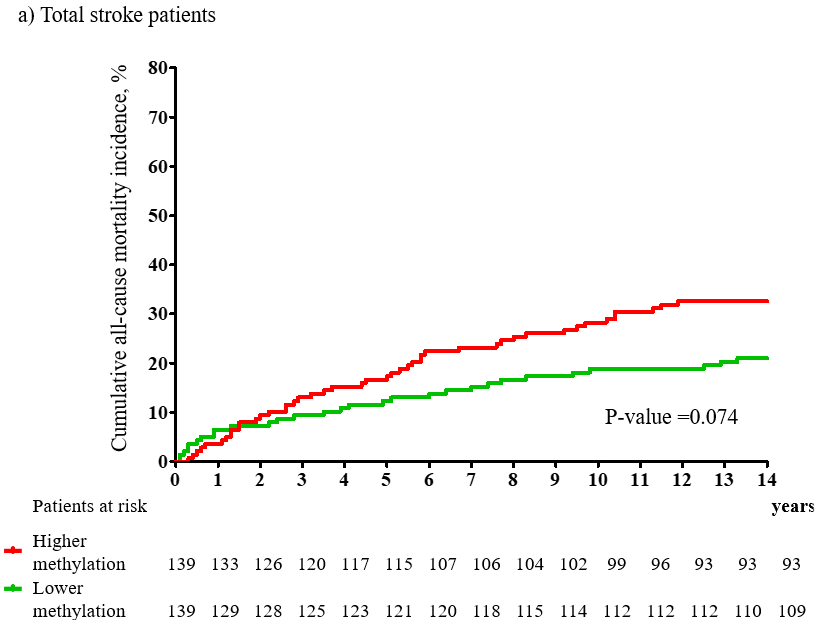

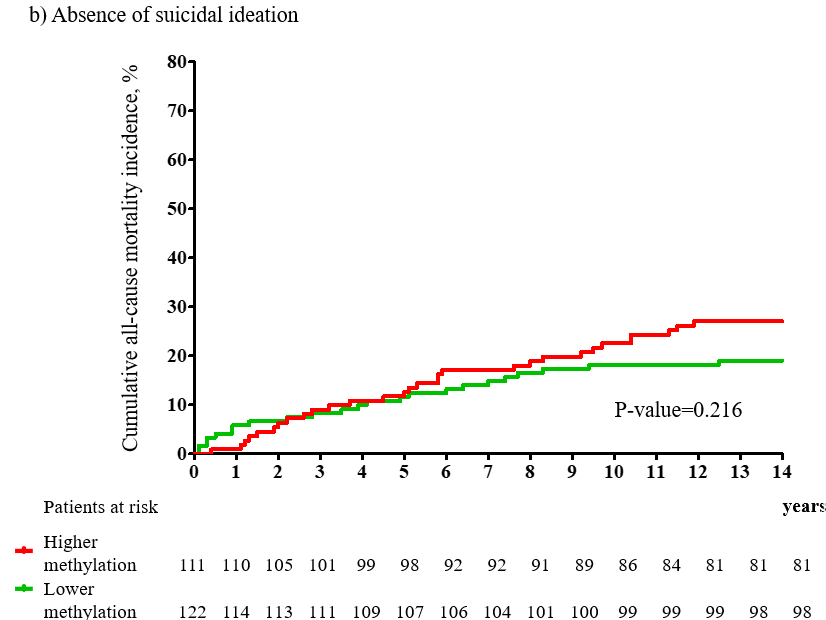

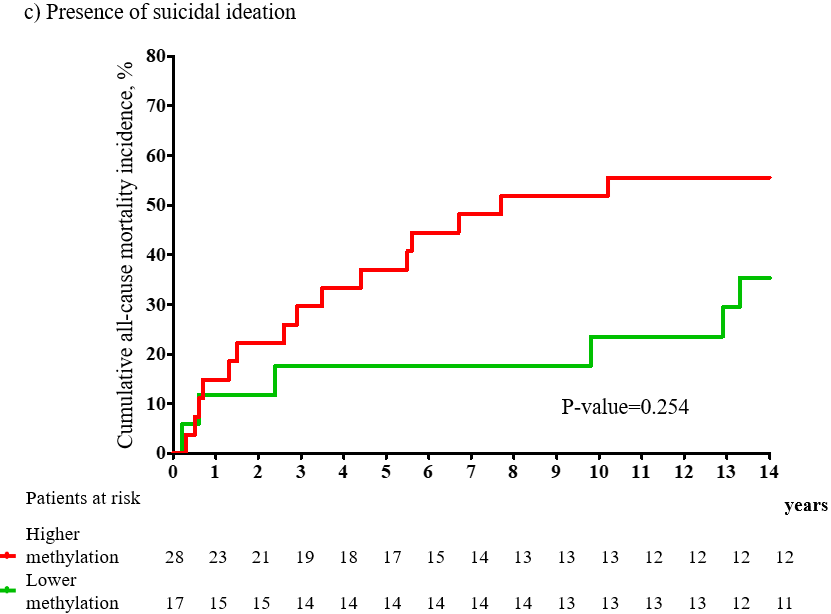


5) CpG 6 The interaction effect between methylation status and SI on composite CCVEs was not significant (*p* =0.273)


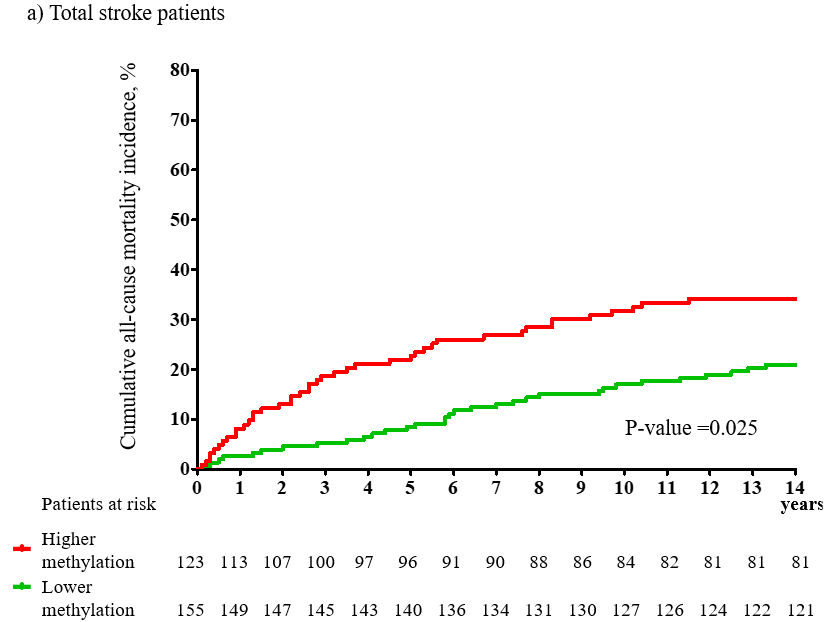

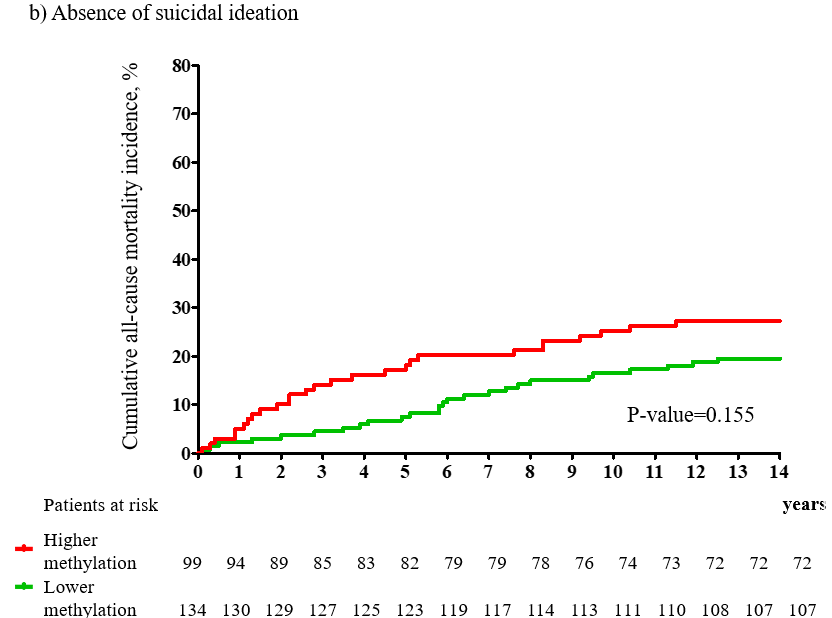

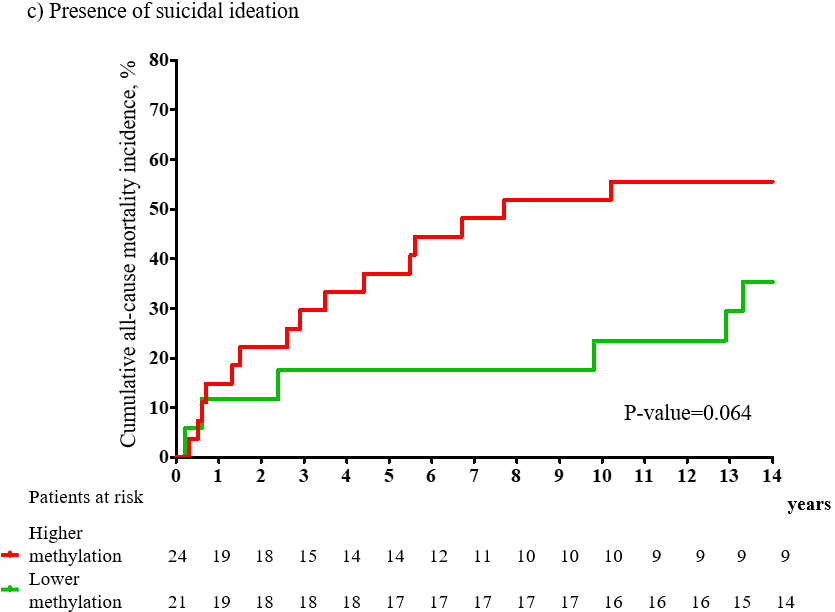


6) CpG 7: The interaction effect between methylation status and SI on composite CCVEs was not significant (*p* =0.212)


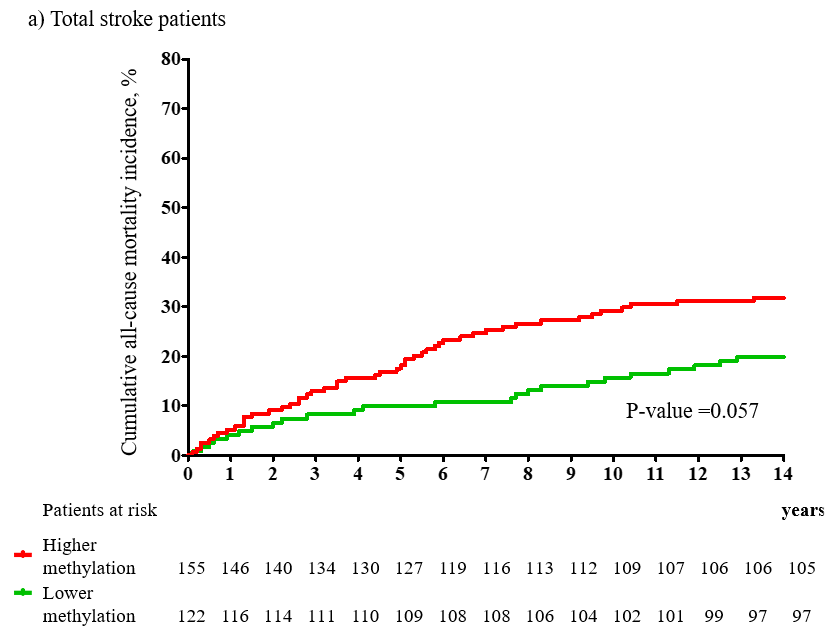

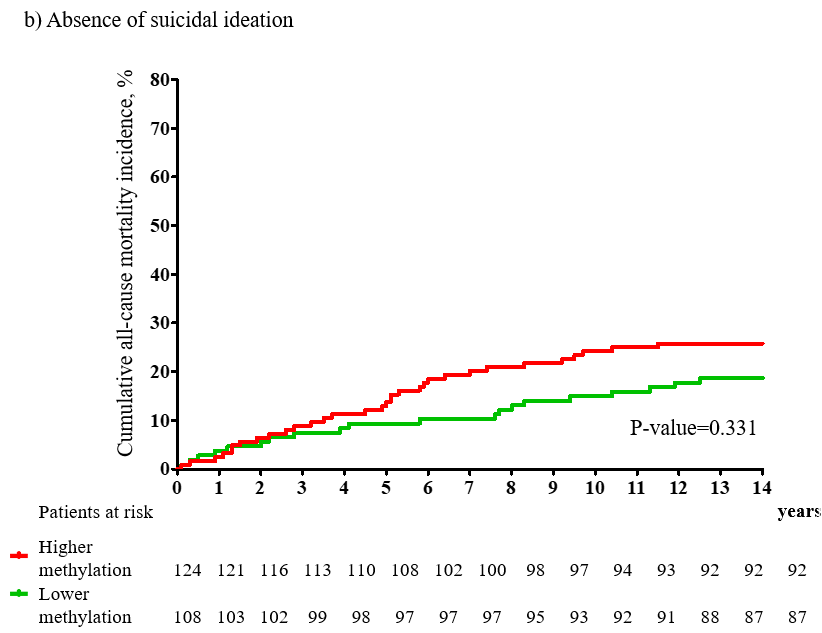

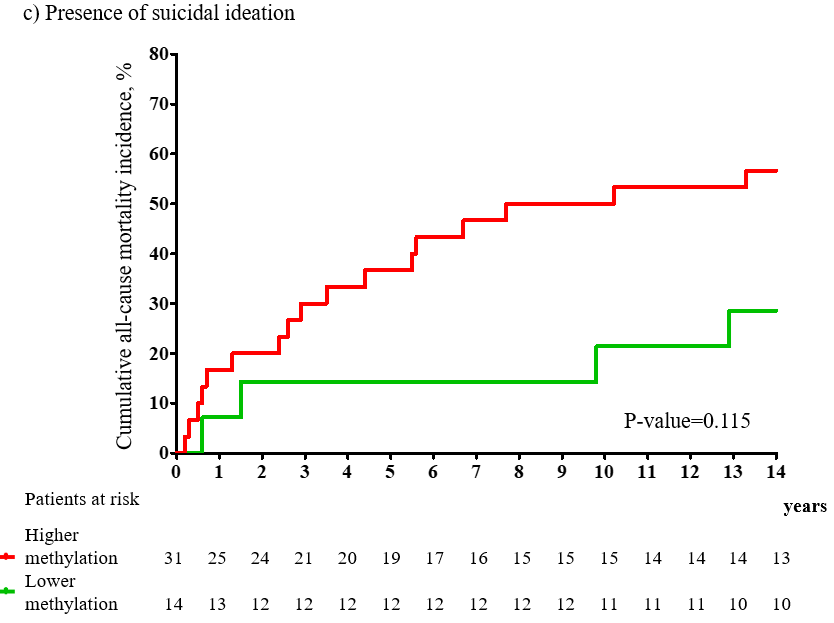


| **Table S1. Number of cerebro-cardiovascular events during the follow-up period by baseline suicidal ideation status and the average *SLC6A4* methylation value** | | | | | |
| --- | --- | --- | --- | --- | --- |
|  | Average methylation value at baseline | |  | Suicidal ideation status at baseline | |
| Long-term outcome | Lower methylation  (n=138) | Higher methylation (n=140) |  | Absence  (n=233) | Presence  (n=45) |
| Recurrent stroke | 19 | 33 |  | 35 | 28 |
| Myocardial infarction | 4 | 12 |  | 14 | 2 |
| Vascular death | 6 | 13 |  | 12 | 7 |
| Cerebro-cardiovascular events (composite) | 25 | 51 |  | 54 | 22 |
|  | | | |  |  |

| **Table S2 *SLC6A4* methylation percentage values at baseline (N = 278).** | | |
| --- | --- | --- |
|  | Median (interquartile range) | Mean (standard deviation) |
| CpG average | 11.1 (7.7-16.7) | 13.9 (11.7) |
| CpG1 | 12.0 (8.0-17.0) | 14.6 (9.9) |
| CpG2 | 16.0 (11.0-25.0) | 20.1 (13.7) |
| CpG3 | 4.0 (0.0-10.3) | 7.6 (11.2) |
| CpG4 | 10.0 (2.0-15.0) | 10.6 (7.5) |
| CpG5 | 10.5 (7.0-16.0) | 15.1 (15.5) |
| CpG6 | 13.0 (9.0-17.0) | 16.0 (15.2) |
| CpG7 | 12.0 (0.0-16.0) | 13.7 (15.1) |

| **Table S3. Baseline characteristics according to the average *SLC6A4* methylation value at 2 weeks after stroke** | | | | | |
| --- | --- | --- | --- | --- | --- |
|  | Total sample  (N=278) | Lower methylation  (N=138) | Higher methylation  (N=140) | Statistical coefficient | *p* value^*^ |
| Age, mean (SD) years | 64.4 (9.5) | 64.1 (9.5) | 64.7 (9.7) | t=-0.504 | 0.615 |
| Gender, N (%) male | 164 (59.0) | 80 (58.0) | 84 (60.0) | χ^2^=0.118 | 0.731 |
| Education, mean (SD) years | 8.4 (5.1) | 8.2 (5.0) | 8.6 (5.2) | t=-0.556 | 0.579 |
| Marital status, N (%) married | 209 (75.2) | 100(72.5) | 109(77.9) | χ^2^=1.083 | 0.298 |
| Current occupation, N (%) have | 123 (44.2) | 60 (43.5) | 63 (45.0) | χ^2^=0.065 | 0.798 |
| DSM-IV depressive disorders | 77 (27.7) | 31 (22.5) | 46 (32.9) | χ^2^=3.749 | 0.053 |
| Cardiac disease | 26 (9.4) | 12 (8.7) | 14 (10.0) | χ^2^=0.139 | 0.709 |
| Previous stroke | 27 (9.7) | 15 (10.9) | 12 (8.6) | χ^2^=0.419 | 0.518 |
| Stroke hemisphere, N (%) |  |  |  |  |  |
| Left | 135 (48.6) | 68 (49.3) | 67 (47.9) | χ^2^=0.401 | 0.819 |
| Right | 133 (47.8) | 66 (47.8) | 67 (47.9) |  |  |
| Bilateral | 10 (3.6) | 4 (2.9) | 6 (4.3) |  |  |
| Stroke location, N (%) |  |  |  |  |  |
| Anterior | 161 (57.9) | 85 (61.6) | 76 (54.3) | χ^2^=2.481 | 0.289 |
| Posterior | 90 (32.4) | 43 (31.2) | 47 (33.6) |  |  |
| Both | 27 (9.7) | 10 (7.2) | 17 (12.1) |  |  |
| NIHSS, mean (SD) score | 3.4 (3.2) | 3.7 (3.3) | 3.2 (3.0) | t=1.208 | 0.228 |
| ^*^ *p* -values using t-tests, χ^2^ tests and Fisher’s exact test as appropriate.  NIHSS, National Institutes of Health Stroke Scale DSM-IV: Diagnostic and Statistical Manual of Mental Disorders, 4^th^ edition | | | | | |

| **Table S4.** **Baseline characteristics according to suicidal ideation status at 2 weeks of stroke.** | | | | | |
| --- | --- | --- | --- | --- | --- |
|  | Total sample  (N=278) | Absent suicidal ideation  (N=233) | Present suicidal ideation  (N=45) | Statistical coefficient | *p* -value^*^ |
| Age, mean (SD) years | 64.4 (9.5) | 64.5 (9.4) | 64.0 (10.3) | t=0.278 | 0.781 |
| Gender, N (%) male | 164 (59.0) | 141 (60.5) | 23 (51.1) | χ^2^=1.379 | 0.240 |
| Education, mean (SD) years | 8.4 (5.1) | 8.5 (5.1) | 7.9 (4.9) | t=0.730 | 0.466 |
| Marital status, N (%) married | 209 (75.2) | 178(76.4) | 31(68.9) | χ^2^=1.139 | 0.286 |
| Current occupation, N (%) have | 123 (44.2) | 106 (45.5) | 17 (37.8) | χ^2^=0.910 | 0.340 |
| DSM-IV depressive disorders | 77 (27.7) | 40 (17.2) | 37 (82.2) | χ^2^=79.705 | **<0.001** |
| Cardiac disease | 26 (9.4) | 24 (10.3) | 2 (4.4) | χ^2^=1.526 | 0.275 |
| Previous stroke | 27 (9.7) | 20 (8.6) | 7 (15.6) | χ^2^=2.091 | 0.148 |
| Stroke hemisphere, N (%) |  |  |  |  |  |
| Left | 135 (48.6) | 114 (48.9) | 21 (46.7) | χ^2^=1.462 | 0.482 |
| Right | 133 (47.8) | 112 (48.1) | 21 (36.7) |  |  |
| Bilateral | 10 (3.6) | 7 (3.0) | 3 (6.7) |  |  |
| Stroke location, N (%) |  |  |  |  |  |
| Anterior | 161 (57.9) | 134 (57.5) | 27 (60.0) | χ^2^=2.971 | 0.226 |
| Posterior | 90 (32.4) | 79 (33.9) | 11 (24.4) |  |  |
| Both | 27 (9.7) | 20 (8.6) | 7 (15.6) |  |  |
| NIHSS, mean (SD) score | 3.4 (3.2) | 3.3 (3.1) | 4.3 (3.6) | t=-2.016 | **0.045** |
| ^*^*p*-values using t-tests, χ^2^ tests and Fisher’s exact test as appropriate.  Bold represents statistical significance (*p* -value < 0.05).  NIHSS, National Institutes of Health Stroke Scale; DSM-IV: Diagnostic and Statistical Manual of Mental Disorders, 4^th^ edition | | | | | |

| **Table S5. Association of CpG 1 methylation status with long-term stroke outcomes (cumulative incidence, %) in the overall cohort and according to suicidal ideation status** | | | | | | | | |
| --- | --- | --- | --- | --- | --- | --- | --- | --- |
| Event | Patients group | Methylation type | Patients Number | Events,  N (%) | Unadjusted  HR (95% CI) | Adjusted^a^ | | *p* -value for interaction^b^ |
|  |  |  |  |  |  | HR (95% CI) | *p* -value |  |
| composite | All patients | Lower | 145 | 29 (20.0) | Ref | Ref |  |  |
|  |  | Higher | 133 | 47 (35.3) | 2.03 (1.28-3.22) | 1.88(1.17-3.00) | **0.009** |  |
|  | SI |  |  |  |  |  |  |  |
|  | absence | Lower | 125 | 24 (19.2) | Ref | Ref |  | 0.066 |
|  |  | Higher | 108 | 30 (27.8) | 1.54 (0.90-2.64) | 1.47(0.85-2.53) | 0.165 |  |
|  | presence | Lower | 20 | 5 (25.0) | Ref | Ref |  |  |
|  |  | Higher | 25 | 17 (68.0) | 4.50 (1.65-12.29) | 4.24(1.42-14.71) | **0.010** |  |
| Recurrent | All patients | Lower | 145 | 21 (14.5) | Ref | Ref |  |  |
| stroke |  | Higher | 133 | 31 (23.3) | 1.81 (1.04-3.15) | 1.77 (1.01-3.11) | **0.046** |  |
|  | SI |  |  |  |  |  |  |  |
|  | absence | Lower | 125 | 17 (13.6) | Ref | Ref |  | 0.100 |
|  |  | Higher | 108 | 18 (16.7) | 1.29 (0.66-2.50) | 1.34 (0.68-2.62) | 0.396 |  |
|  | presence | Lower | 20 | 4 (20.0) | Ref | Ref |  |  |
|  |  | Higher | 25 | 13 (52.0) | 4.15 (1.34-12.85) | 4.59 (1.28-16.44) | **0.019** |  |
| Myocardial | All patients | Lower | 145 | 5 (3.4) | Ref | Ref |  |  |
| infarction |  | Higher | 133 | 11 (8.3) | 2.50 (0.87-7.21) | 2.07 (0.71-6.02) | 0.183 |  |
|  | SI |  |  |  |  |  |  |  |
|  | absence | Lower | 125 | 4 (3.2) | Ref | Ref |  | 0.356 |
|  |  | Higher | 108 | 10 (9.3) | 2.93 (0.92-9.34) | 2.33 (0.72-7.59) | 0.161 |  |
|  | presence | Lower | 20 | 1 (5.0) | Ref | Ref |  |  |
|  |  | Higher | 25 | 1 (4.0) | 0.89 (0.06-14.27) | 0.04 (0.00-6.92) | 0.225 |  |
| Vascular | All patients | Lower | 145 | 6 (4.1) | Ref | Ref |  |  |
| death |  | Higher | 133 | 13 (9.8) | 2.49 (0.95-6.56) | 2.22(0.83-5.89) | 0.111 |  |
|  | SI |  |  |  |  |  |  |  |
|  | absence | Lower | 125 | 5 (4.0) | Ref | Ref |  | 0.311 |
|  |  | Higher | 108 | 7 (6.5) | 1.68 (0.53-5.31) | 1.46 (0.46-4.67) | 0.525 |  |
|  | presence | Lower | 20 | 1 (5.0) | Ref | Ref |  |  |
|  |  | Higher | 25 | 6 (24.0) | 5.25 (0.63-43.64) | 5.80 (0.63-53.11) | 0.120 |  |
| The higher and lower methylation were classified using the median value.  HR (95% CI) were calculated using Cox proportional hazards models. Bold represents statistical significance (*p* -value < 0.05)  ^a^Adjusted for age, NIHSS score, previous history of stroke, presence of cardiac disease and DSM-IV depression at 2 weeks after stroke  ^b^The interactive effect between *SLC6A4* methylation value and suicidal ideation on CCVEs were calculated in the same adjusted model.  HR, Hazard ratios; CI, confidence interval; CCVEs; Cerebro-cardiovascular events, SI, suicidal ideation; NIHSS, National Institutes of Health Stroke Scale; DSM-IV, Diagnostic and Statistical Manual of Mental Disorders, 4^th^ edition | | | | | | | | |

| **Table S6.** **Association of CpG 2 methylation status with long-term stroke outcomes (cumulative incidence, %) in the overall cohort and according to suicidal ideation status** | | | | | | | | |
| --- | --- | --- | --- | --- | --- | --- | --- | --- |
| Event | Patients group | Methylation type | Patient numbers | Events,  N (%) | Unadjusted  HR (95% CI) | Adjusted^a^ | | *p* -value for interaction^b^ |
|  |  |  |  |  |  | HR (95% CI) | *p* -value |  |
| composite | All patients | Lower | 141 | 25 (17.7) | Ref | Ref |  |  |
|  |  | Higher | 137 | 51 (37.2) | 2.39 (1.48-3.86) | 2.22(1.36-3.60) | **0.001** |  |
|  | SI |  |  |  |  |  |  |  |
|  | absence | Lower | 119 | 18 (15.1) | Ref | Ref |  | 0.552 |
|  |  | Higher | 114 | 36 (31.6) | 2.29 (1.30-4.04) | 2.16(1.22-3.83) | **0.008** |  |
|  | presence | Lower | 22 | 7 (31.8) | Ref | Ref |  |  |
|  |  | Higher | 23 | 15 (65.2) | 2.72 (1.10-6.73) | 2.36(0.88-6.31) | **0.009** |  |
| Recurrent | All patients | Lower | 141 | 19 (13.5) | Ref | Ref |  |  |
| stroke |  | Higher | 137 | 33 (24.1) | 1.97 (1.12-3.48) | 1.94 (1.09-3.44) | **0.024** |  |
|  | SI |  |  |  |  |  |  |  |
|  | absence | Lower | 119 | 13 (10.9) | Ref | Ref |  | 0.699 |
|  |  | Higher | 114 | 22 (19.3) | 1.87 (0.94-3.72) | 1.91 (0.96-3.83) | 0.067 |  |
|  | presence | Lower | 22 | 6 (27.3) | Ref | Ref |  |  |
|  |  | Higher | 23 | 11 (47.8) | 2.27 (0.83-6.22) | 2.18 (0.72-6.66) | 0.170 |  |
| Myocardial | All patients | Lower | 141 | 4 (2.8) | Ref | Ref |  |  |
| infarction |  | Higher | 137 | 12 (8.8) | 3.22 (1.04-9.99) | 2.68 (0.86-8.40) | 0.091 |  |
|  | SI |  |  |  |  |  |  |  |
|  | absence | Lower | 119 | 3 (2.5) | Ref | Ref |  | 0.345 |
|  |  | Higher | 114 | 11 (9.6) | 3.88 (1.08-13.90) | 3.06 (0.84-11.16) | 0.091 |  |
|  | presence | Lower | 22 | 1 (4.5) | Ref | Ref |  |  |
|  |  | Higher | 23 | 1 (4.3) | 1.08 (0.07-17.24) | 0.28 (0.00-22.22) | 0.565 |  |
| Vascular | All patients | Lower | 141 | 7 (5.0) | Ref | Ref |  |  |
| death |  | Higher | 137 | 12 (8.8) | 1.84 (0.72-4.67) | 1.63 (0.64-4.19) | 0.307 |  |
|  | SI |  |  |  |  |  |  |  |
|  | absence | Lower | 119 | 6 (5.0) | Ref | Ref |  | 0.120 |
|  |  | Higher | 114 | 6 (5.3) | 1.07 (0.34-3.31) | 0.93 (0.29-2.94) | 0.903 |  |
|  | presence | Lower | 22 | 1 (4.5) | Ref | Ref |  |  |
|  |  | Higher | 23 | 6 (26.1) | 6.36 (0.77-52.87) | 6.07 (0.67-54.84) | 0.108 |  |
| The higher and lower methylation were classified using the median value.  HR (95% CI) were calculated using Cox proportional hazards models. Bold represents statistical significance (*p* -value < 0.05).  ^a^Adjusted for age, NIHSS score, previous history of stroke, presence of cardiac disease and DSM-IV depression at 2 weeks after stroke  ^b^The interactive effect between *SLC6A4* methylation value and suicidal ideation on CCVEs were calculated in the same adjusted model.  HR, Hazard ratios; CI, confidence interval; CCVEs; Cerebro-cardiovascular events, SI, suicidal ideation; NIHSS, National Institutes of Health Stroke Scale; DSM-IV, Diagnostic and Statistical Manual of Mental Disorders, 4^th^ edition | | | | | | | | |

| **Table S7** **Association of CpG 3 methylation status with long-term stroke outcomes (cumulative incidence, %) in the overall cohort and according to suicidal ideation status** | | | | | | | | |
| --- | --- | --- | --- | --- | --- | --- | --- | --- |
| Event | Patients group | Methylation type | Patient numbers | Events,  N (%) | Unadjusted  HR (95% CI) | Adjusted^a^ | | *p* -value for interaction^b^ |
|  |  |  |  |  |  | HR (95% CI) | *p* -value |  |
| composite | All patients | Lower | 143 | 34 (23.8) | Ref | Ref |  |  |
|  |  | Higher | 135 | 42 (31.1) | 1.36 (0.87-2.14) | 1.36 (0.87-2.15) | 0.180 |  |
|  | SI |  |  |  |  |  |  |  |
|  | absence | Lower | 123 | 27 (22.0) | Ref | Ref |  | 0.362 |
|  |  | Higher | 110 | 27 (24.5) | 1.14 (0.67-1.94) | 1.22 (0.71-2.09) | 0.479 |  |
|  | presence | Lower | 20 | 7 (35.0) | Ref | Ref |  |  |
|  |  | Higher | 25 | 15 (60.0) | 1.95 (0.79-4.78) | 2.18 (0.77-6.15) | 0.141 |  |
| Recurrent | All patients | Lower | 143 | 22 (15.4) | Ref | Ref |  |  |
| stroke |  | Higher | 135 | 30 (22.2) | 1.50 (0.86-2.60) | 1.48 (0.85-2.57) | 0.165 |  |
|  | SI |  |  |  |  |  |  |  |
|  | absence | Lower | 123 | 15 (12.2) | Ref | Ref |  | 0.729 |
|  |  | Higher | 110 | 20 (18.2) | 1.53 (0.79-2.99) | 1.52 (0.77-3.01) | 0.228 |  |
|  | presence | Lower | 20 | 7 (35.0) | Ref | Ref |  |  |
|  |  | Higher | 25 | 17 (68.0) | 1.28(0.49-3.38) | 1.39 (0.46-4.20) | 0.562 |  |
| Myocardial | All patients | Lower | 143 | 10 (7.0) | Ref | Ref |  |  |
| infarction |  | Higher | 135 | 6 (4.4) | 1.33 (0.81-2.18) | 0.62 (0.22-1.70) | 0.351 |  |
|  | SI |  |  |  |  |  |  |  |
|  | absence | Lower | 123 | 10 (8.1) | Ref | Ref |  | NA |
|  |  | Higher | 110 | 4 (3.6) | 0.44 (0.14-1.39) | 0.51 (0.16-1.65) | 0.261 |  |
|  | presence | Lower | 20 | 0 (0) | Ref | Ref |  |  |
|  |  | Higher | 25 | 2 (8.0) | 53.70 (0.00-5136116.67) | 130938.48 (0.00-1.12E+269) | 0.970 |  |
| Vascular | All patients | Lower | 143 | 9 (6.3) | Ref | Ref |  |  |
| death |  | Higher | 135 | 10 (7.4) | 1.17 (0.48-2.88) | 1.12 (0.46-2.78) | 0.218 |  |
|  | SI |  |  |  |  |  |  |  |
|  | absence | Lower | 123 | 7 (5.7) | Ref | Ref |  | 0.492 |
|  |  | Higher | 110 | 5 (4.5) | 0.78 (0.25-2.46) | 0.85 (0.27-2.71) | 0.785 |  |
|  | presence | Lower | 20 | 2 (20.0) | Ref | Ref |  |  |
|  |  | Higher | 25 | 5 (20.0) | 2.01 (0.39-10.37) | 2.86 (0.36-22.40) | 0.318 |  |
| The higher and lower methylation were classified using the median value.  HR (95% CI) were calculated using Cox proportional hazards models. Bold represents statistical significance (*p* -value < 0.05).  ^a^Adjusted for age, NIHSS score, previous history of stroke, presence of cardiac disease and DSM-IV depression at 2 weeks after stroke  ^b^The interactive effect between *SLC6A4* methylation value and suicidal ideation on CCVEs were calculated in the same adjusted model.  HR, Hazard ratios; CI, confidence interval; CCVEs; Cerebro-cardiovascular events, SI, suicidal ideation; NIHSS, National Institutes of Health Stroke Scale; DSM-IV, Diagnostic and Statistical Manual of Mental Disorders, 4^th^ edition | | | | | | | | |

| **Table S8.** **Association of CpG 4 methylation status with long-term stroke outcomes (cumulative incidence, %) in the overall cohort and according to suicidal ideation status** | | | | | | | | |
| --- | --- | --- | --- | --- | --- | --- | --- | --- |
| Event | Patients group | Methylation type | Patient numbers | Events,  N (%) | Unadjusted  HR (95% CI) | Adjusted^a^ | | *p* -value for interaction^b^ |
|  |  |  |  |  |  | HR (95% CI) | *p* -value |  |
| composite | All patients | Lower | 143 | 29(20.3) | Ref | Ref |  |  |
|  |  | Higher | 135 | 47(34.8) | 1.94 (1.22-3.08) | 1.87(1.17-2.99) | **0.009** |  |
|  | SI |  |  |  |  |  |  |  |
|  | absence | Lower | 123 | 25 (20.3) | Ref | Ref |  | **0.012** |
|  |  | Higher | 110 | 29 (26.4) | 1.36 (0.79-2.32) | 1.33(0.78-2.29) | 0.295 |  |
|  | presence | Lower | 20 | 4 (20.0) | Ref | Ref |  |  |
|  |  | Higher | 25 | 18 (72.0) | 6.00 (2.00-17.97) | 6.15(1.85-20.49) | **0.003** |  |
| Recurrent | All patients | Lower | 143 | 19 (13.3) | Ref | Ref |  |  |
| stroke |  | Higher | 135 | 33 (24.4) | 2.05 (1.17-3.62) | 2.05 (1.16-3.62)) | **0.014** |  |
|  | SI |  |  |  |  |  |  |  |
|  | absence | Lower | 123 | 16 (13.0) | Ref | Ref |  | **0.039** |
|  |  | Higher | 110 | 19 (17.3) | 1.38 (0.71-2.68) | 1.41 (0.72-2.75) | 0.319 |  |
|  | presence | Lower | 20 | 3 (15.0) | Ref | Ref |  |  |
|  |  | Higher | 25 | 14 (56.0) | 6.19 (1.75-21.92) | 7.75 (1.84-32.61) | **0.005** |  |
| Myocardial | All patients | Lower | 143 | 8 (5.6) | Ref | Ref |  |  |
| infarction |  | Higher | 135 | 8 (5.9) | 1.08 (0.41-2.87) | 0.90 (0.33-2.46) | 0.842 |  |
|  | SI |  |  |  |  |  |  |  |
|  | absence | Lower | 123 | 7 (5.7) | Ref | Ref |  | 0.766 |
|  |  | Higher | 110 | 7 (6.4) | 1.10 (0.39-3.14) | 0.87 (0.29-2.57) | 0.799 |  |
|  | presence | Lower | 20 | 1 (5.0) | Ref | Ref |  |  |
|  |  | Higher | 25 | 1 (4.0) | 0.89 (0.06-14.27) | 0.26 (0.00-16.04) | 0.525 |  |
| Vascular | All patients | Lower | 143 | 8 (5.6) | Ref | Ref |  |  |
| death |  | Higher | 135 | 11 (8.1) | 1.50 (0.60-3.71) | 1.38 (0.55-3.45) | 0.496 |  |
|  | SI |  |  |  |  |  |  |  |
|  | absence | Lower | 123 | 7 (5.7) | Ref | Ref |  | 0.120 |
|  |  | Higher | 110 | 5 (4.5) | 0.80 (0.25-2.51) | 0.72 (0.22-2.30) | 0.575 |  |
|  | presence | Lower | 20 | 1 (5.0) | Ref | Ref |  |  |
|  |  | Higher | 25 | 6 (24.0) | 5.25 (0.63-43.64) | 5.59 (0.61-51.13) | 0.128 |  |
| The higher and lower methylation were classified using the median value.  HR (95% CI) were calculated using Cox proportional hazards models. Bold represents statistical significance (*p* -value < 0.05).  ^a^Adjusted for age, NIHSS score, previous history of stroke, presence of cardiac disease and DSM-IV depression at 2 weeks after stroke  ^b^The interactive effect between *SLC6A4* methylation value and suicidal ideation on CCVEs were calculated in the same adjusted model.  HR, Hazard ratios; CI, confidence interval; CCVEs; Cerebro-cardiovascular events, SI, suicidal ideation; NIHSS, National Institutes of Health Stroke Scale; DSM-IV, Diagnostic and Statistical Manual of Mental Disorders, 4^th^ edition | | | | | | | | |

| **Table S9. Association of CpG 5 methylation status with long-term stroke outcomes (cumulative incidence, %) in the overall cohort and according to suicidal ideation status** | | | | | | | | |
| --- | --- | --- | --- | --- | --- | --- | --- | --- |
| Event | Patients group | Personality type | Patient numbers | Events,  N (%) | Unadjusted  HR (95% CI) | Adjusted^a^ | | *p* -value for interaction^b^ |
|  |  |  |  |  |  | HR (95% CI) | *p* -value |  |
| composite | All patients | Lower | 139 | 30 (21.6) | Ref | Ref |  |  |
|  |  | Higher | 139 | 46 (33.1) | 1.63 (1.03-2.59) | 1.53(0.96-2.45) | 0.074 |  |
|  | SI |  |  |  |  |  |  |  |
|  | absence | Lower | 122 | 24 (19.7) | Ref | Ref |  | 0.586 |
|  |  | Higher | 111 | 30 (27.0) | 1.39 (0.82-2.39) | 1.41(0.82-2.44) | 0.216 |  |
|  | presence | Lower | 17 | 6 (35.3) | Ref | Ref |  |  |
|  |  | Higher | 28 | 16 (57.1) | 2.09 (0.81-5.37) | 1.92(0.63-5.92) | 0.254 |  |
| Recurrent | All patients | Lower | 139 | 22 (15.8) | Ref | Ref |  |  |
| stroke |  | Higher | 139 | 30 (21.6) | 1.41 (0.82-2.45) | 1.39 (0.79-2.44) | 0.254 |  |
|  | SI |  |  |  |  |  |  |  |
|  | absence | Lower | 122 | 16 (13.1) | Ref | Ref |  | 0.915 |
|  |  | Higher | 111 | 19 (17.1) | 1.29 (0.67-2.52) | 1.40 (0.71-2.75) | 0.333 |  |
|  | presence | Lower | 17 | 6 (35.3) | Ref | Ref |  |  |
|  |  | Higher | 28 | 11 (39.3) | 1.42 (0.52-3.88) | 1.39 (0.42-4.59) | 0.588 |  |
| Myocardial | All patients | Lower | 139 | 5 (3.6) | Ref | Ref |  |  |
| infarction |  | Higher | 139 | 11 (7.9) | 2.26 (0.79-6.52) | 2.01 (0.69-5.85) | 0.185 |  |
|  | SI |  |  |  |  |  |  |  |
|  | absence | Lower | 122 | 5 (4.1) | Ref | Ref |  | NA |
|  |  | Higher | 111 | 9 (8.1) | 1.96 (0.66-5.85) | 1.81 (0.60-5.45) | 0.290 |  |
|  | presence | Lower | 17 | 0 (0.0) | Ref | Ref |  |  |
|  |  | Higher | 28 | 2 (7.1) | 46.38 (0.00-5625545.78) | 280107.03 (0.00-NA) | 0.980 |  |
| Vascular | All patients | Lower | 139 | 8 (5.8) | Ref | Ref |  |  |
| death |  | Higher | 139 | 11 (7.9) | 1.41 (0.57-3.50) | 1.34 (0.84-2.13) | 0.218 |  |
|  | SI |  |  |  |  |  |  |  |
|  | absence | Lower | 122 | 8 (6.6) | Ref | Ref |  | NA |
|  |  | Higher | 111 | 4 (3.6) | 0.54 (0.16-1.80) | 0.89 (0.24-3.35) | 0.865 |  |
|  | presence | Lower | 17 | 0 (0.0) | Ref | Ref |  |  |
|  |  | Higher | 28 | 7 (25.0) | 47.19 (0.09-24219.58) | 329610.79 (0.00-6.83e+238) | 0.963 |  |
| The higher and lower methylation were classified using the median value.  HR (95% CI) were calculated using Cox proportional hazards models. Bold represents statistical significance (*p* -value < 0.05).  ^a^Adjusted for age, NIHSS score, previous history of stroke, presence of cardiac disease and DSM-IV depression at 2 weeks after stroke  ^b^The interactive effect between *SLC6A4* methylation value and suicidal ideation on CCVEs were calculated in the same adjusted model.  HR, Hazard ratios; CI, confidence interval; CCVEs; Cerebro-cardiovascular events, SI, suicidal ideation; NIHSS, National Institutes of Health Stroke Scale; DSM-IV, Diagnostic and Statistical Manual of Mental Disorders, 4^th^ edition | | | | | | | | |

| **Table S10.** **Association of CpG 6 methylation status with long-term stroke outcomes (cumulative incidence, %) in the overall cohort and according to suicidal ideation status** | | | | | | | | |
| --- | --- | --- | --- | --- | --- | --- | --- | --- |
| Event | Patients group | Methylation type | Patient numbers | Events,  N (%) | Unadjusted  HR (95% CI) | Adjusted^a^ | | *p* -value for interaction^b^ |
|  |  |  |  |  |  | HR (95% CI) | *p* -value |  |
| composite | All patients | Lower | 155 | 34 (21.9) | Ref | Ref |  |  |
|  |  | Higher | 123 | 42 (34.1) | 1.80 (1.14-2.83) | 1.69(0.99-2.63) | **0.025** |  |
|  | SI |  |  |  |  |  |  |  |
|  | absence | Lower | 134 | 27 (20.1) | Ref | Ref |  | 0.273 |
|  |  | Higher | 99 | 27 (27.3) | 1.49 (0.87-2.54) | 1.48(0.86-2.53) | 0.155 |  |
|  | presence | Lower | 21 | 7 (33.3) | Ref | Ref |  |  |
|  |  | Higher | 24 | 15 (62.5) | 2.66 (1.07-6.60) | 2.65(0.95-7.44) | 0.064 |  |
| Recurrent | All patients | Lower | 155 | 22 (14.2) | Ref | Ref |  |  |
| stroke |  | Higher | 123 | 30 (24.4) | 1.94 (1.12-3.37) | 1.90 (1.09-3.32) | **0.024** |  |
|  | SI |  |  |  |  |  |  |  |
|  | absence | Lower | 134 | 15 (11.2) | Ref | Ref |  | 0.727 |
|  |  | Higher | 99 | 20 (20.2) | 1.95 (1.00-3.82) | 2.01 (1.03-3.95) | **0.041** |  |
|  | presence | Lower | 21 | 7 (33.3) | Ref | Ref |  |  |
|  |  | Higher | 24 | 10 (41.7) | 1.73 (0.65-4.63) | 1.87 (0.61-5.71) | 0.271 |  |
| Myocardial | All patients | Lower | 155 | 7 (4.5) | Ref | Ref |  |  |
| infarction |  | Higher | 123 | 9 (7.3) | 1.66 (0.62-4.47) | 1.44 (0.53-3.94) | 0.474 |  |
|  | SI |  |  |  |  |  |  |  |
|  | absence | Lower | 134 | 7 (5.2) | Ref | Ref |  | NA |
|  |  | Higher | 99 | 7 (7.1) | 1.35 (0.47-3.84) | 1.18 (0.41-3.43) | 0.764 |  |
|  | presence | Lower | 21 | 0 (0.0) | Ref | Ref |  |  |
|  |  | Higher | 24 | 2 (8.3) | 61.02 (0.00-5348480.92) | 105361.22 (0.00-1.64E+277) | 0.971 |  |
| Vascular | All patients | Lower | 155 | 10 (6.5) | Ref | Ref |  |  |
| death |  | Higher | 123 | 9 (7.3) | 1.17 (0.47-2.87) | 1.00 (0.40-2.50) | 0.994 |  |
|  | SI |  |  |  |  |  |  |  |
|  | absence | Lower | 134 | 9 (6.7) | Ref | Ref |  | **0.038** |
|  |  | Higher | 99 | 3 (3.0) | 0.45 (0.12-1.66) | 0.42 (0.11-1.57) | 0.196 |  |
|  | presence | Lower | 21 | 1 (4.8) | Ref | Ref |  |  |
|  |  | Higher | 24 | 6 (25.0) | 5.70 (0.69-47.39) | 3.41 (0.38-30.35) | 0.271 |  |
| The higher and lower methylation were classified using the median value.  HR (95% CI) were calculated using Cox proportional hazards models. Bold represents statistical significance (*p* -value < 0.05).  ^a^Adjusted for age, NIHSS score, previous history of stroke, presence of cardiac disease and DSM-IV depression at 2 weeks after stroke  ^b^The interactive effect between *SLC6A4* methylation value and suicidal ideation on CCVEs were calculated in the same adjusted model.  HR, Hazard ratios; CI, confidence interval; CCVEs; Cerebro-cardiovascular events, SI, suicidal ideation; NIHSS, National Institutes of Health Stroke Scale; DSM-IV, Diagnostic and Statistical Manual of Mental Disorders, 4^th^ edition | | | | | | | | |

| **Table S11. Association of CpG 7 methylation status with long-term stroke outcomes (cumulative incidence, %) in the overall cohort and according to suicidal ideation status** | | | | | | | | |
| --- | --- | --- | --- | --- | --- | --- | --- | --- |
| Event | Patients group | Methylation type | Patient numbers | Events,  N (%) | Unadjusted  HR (95% CI) | Adjusted^a^ | | *p*-value for interaction^b^ |
|  |  |  |  |  |  | HR (95% CI) | *p* -value |  |
| composite | All patients | Lower | 122 | 25 (20.5) | Ref | Ref |  |  |
|  |  | Higher | 155 | 50 (32.3) | 1.73 (1.07-2.79) | 1.61(0.99-2.63) | 0.057 |  |
|  | SI |  |  |  |  |  |  |  |
|  | absence | Lower | 108 | 21 (19.4) | Ref | Ref |  | 0.212 |
|  |  | Higher | 124 | 32 (25.8) | 1.41 (0.81-2.44) | 1.32(0.75-2.33) | 0.331 |  |
|  | presence | Lower | 14 | 4 (28.6) | Ref | Ref |  |  |
|  |  | Higher | 31 | 18 (58.1) | 2.55 (0.86-7.57) | 2.48(0.80-7.63) | 0.115 |  |
| Recurrent | All patients | Lower | 122 | 17 (13.9) | Ref | Ref |  |  |
| stroke |  | Higher | 155 | 35 (22.6) | 1.75 (0.98-3.12) | 1.67 (0.92-3.00) | 0.090 |  |
|  | SI |  |  |  |  |  |  |  |
|  | absence | Lower | 108 | 13 (12.0) | Ref | Ref |  | 0.778 |
|  |  | Higher | 124 | 22 (17.7) | 1.55 (0.78-3.08) | 1.49 (0.74-2.99) | 0.264 |  |
|  | presence | Lower | 14 | 4 (28.6) | Ref | Ref |  |  |
|  |  | Higher | 31 | 13 (41.9) | 1.80 (0.58-5.54) | 1.84 (0.57-5.93) | 0.309 |  |
| Myocardial | All patients | Lower | 122 | 6 (4.9) | Ref | Ref |  |  |
| infarction |  | Higher | 155 | 9 (5.8) | 1.20 (0.43-3.38) | 1.07 (0.37-3.15) | 0.898 |  |
|  | SI |  |  |  |  |  |  |  |
|  | absence | Lower | 108 | 6 (5.6) | Ref | Ref |  | NA |
|  |  | Higher | 124 | 7 (5.6) | 1.01 (0.34-3.01) | 0.77 (0.23-2.52) | 0.660 |  |
|  | presence | Lower | 14 | 0 (0.0) | Ref | Ref |  |  |
|  |  | Higher | 31 | 2 (6.5) | 36.91 (0.00-9874499.06) | 376103.49 (0.00-NA) | 0.982 |  |
| Vascular | All patients | Lower | 122 | 7 (5.7) | Ref | Ref |  |  |
| death |  | Higher | 155 | 12 (7.7) | 1.39 (0.55-3.52) | 1.25 (0.48-3.28) | 0.648 |  |
|  | SI |  |  |  |  |  |  |  |
|  | absence | Lower | 108 | 6 (5.6) | Ref | Ref |  | 0.293 |
|  |  | Higher | 124 | 6 (4.8) | 0.89 (2.9-2.75) | 0.90 (0.28-2.92) | 0.858 |  |
|  | presence | Lower | 14 | 1 (7.1) | Ref | Ref |  |  |
|  |  | Higher | 31 | 6 (19.4) | 2.82 (0.34-23.46) | 1.94 (0.21-17.47) | 0.556 |  |
| The higher and lower methylation were classified using the median value.  HR (95% CI) were calculated using Cox proportional hazards models. Bold represents statistical significance (*p* -value < 0.05).  ^a^Adjusted for age, NIHSS score, previous history of stroke, presence of cardiac disease and DSM-IV depression at 2 weeks after stroke  ^b^The interactive effect between *SLC6A4* methylation value and suicidal ideation on CCVEs were calculated in the same adjusted model.  HR, Hazard ratios; CI, confidence interval; CCVEs; Cerebro-cardiovascular events, SI, suicidal ideation; NIHSS, National Institutes of Health Stroke Scale; DSM-IV, Diagnostic and Statistical Manual of Mental Disorders, 4^th^ edition | | | | | | | | |
